# Identification of a group of 9-amino-acridines that selectively downregulate regulatory T cell functions through FoxP3

**DOI:** 10.1016/j.isci.2025.111931

**Published:** 2025-01-31

**Authors:** Qian Wei, Håvard Foyn, Johannes Landskron, Shixiong Wang, Inga Hansine Rye, Sigrid S. Skånland, Hege Elisabeth Giercksky Russnes, Jo Klaveness, Rafi Ahmad, Kjetil Taskén

**Affiliations:** 1 Department of Cancer Immunology, Institute for Cancer Research, Oslo University Hospital, 0424 Oslo, Norway; 2 Norwegian Centre for Clinical Cancer Research, MATRIX, Division of Cancer Medicine, Oslo University Hospital, 0424 Oslo, Norway; 3 Centre for Molecular Medicine Norway, Nordic EMBL Partnership, University of Oslo, 0318 Oslo, Norway; 4 Department of Cancer Genetics, Institute for Cancer Research, Oslo University Hospital, 0424 Oslo, Norway; 5 K.G. Jebsen Centre for B Cell Malignancies, Institute of Clinical Medicine, University of Oslo, 0317 Oslo, Norway; 6 Department of Pathology, Division of Laboratory Medicine, Oslo University Hospital, 0424 Oslo, Norway; 7 Institute of Clinical Medicine, University of Oslo, 0318 Oslo, Norway; 8 Department of Pharmacy, University of Oslo, 0371 Oslo, Norway; 9 Department of Biotechnology, University of Inland Norway, 2317 Hamar, Norway

**Keywords:** Immunology, Immune response, Cancer

## Abstract

FoxP3^+^ regulatory T cells (Tregs) are responsible for immune homeostasis by suppressing excessive anti-self-immunity. Tregs facilitate tumor growth by inhibiting anti-tumor immunity. Here, we explored the targeting of FoxP3 as a basis for new immunotherapies. In a high-throughput phenotypic screening of a drug repurposing library using human primary T cells, we identified quinacrine as a FoxP3 downregulator. *In silico* searches based on the structure of quinacrine, testing of sub-libraries of analogs *in vitro,* and validation identified a subset of 9-amino-acridines that selectively abrogated Treg suppressive functions. Mechanistically, these acridines interfered with the DNA-binding activity of FoxP3 and inhibited FoxP3-regulated downstream gene regulation. Release from Treg suppression by 9-amino-acridines increased anti-tumor immune responses both in cancer patient samples and in mice in a syngeneic tumor model. Our study highlights the feasibility of screening for small molecular inhibitors of FoxP3 as an approach to pursuing Treg-based immunotherapy.

## Highlights

• Lack of selectivity has hampered drug development of small-molecule Treg inhibitors
• A cell-based drug screen in human primary T cells identified FoxP3 downregulators
• A group of 9-amino-acridines selectively inhibits Tregs through FoxP3 regulation
• These compounds boost anti-tumor immunity in patient samples and a mouse tumor model

## Graphical abstract

**Figure:**
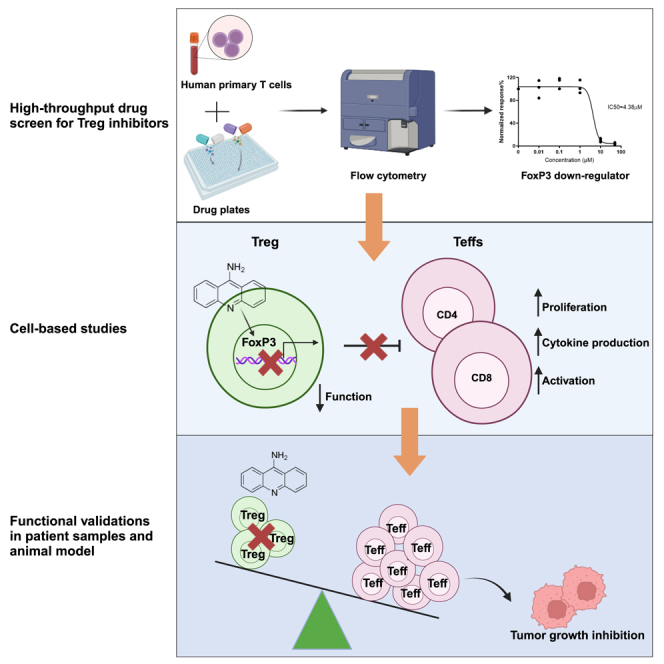

## Introduction

FoxP3^+^ regulatory T cells (Tregs) inhibit the function of effector T cells (Teffs). They are known to control autoimmunity and inhibit anti-viral immune responses and anti-tumor immune responses.^1^^,^^2^ Control of autoimmunity is mainly mediated by naturally occurring Tregs (nTregs) that are educated in the thymus and exist from early life. In contrast, inhibition of anti-viral and anti-tumor immune responses is mainly mediated by adaptive or peripherally induced Tregs (iTregs) formed at sites of immune activation from Teffs due to sustained immune activation.^3^^,^^4^ The formation of iTregs is programmed when T cell activation lasts over prolonged periods and is normally a functional response to turn off immune activation and prevent immunological overshot and tissue damage after the immune system has successfully cleared an infection.^5^ However, in immune-oncology Treg-mediated inhibition of other immune cells in the tumor microenvironment (TME) constitutes an important tumor immune evasion mechanism.^6^^,^^7^ Higher proportions of Tregs in the TME are reported to be related to poor prognosis in many different types of cancers.^8^ It is, therefore, of interest to target and turn off Tregs to boost anti-tumor immune responses.

Despite the substantial evidence of the immunosuppressive function of Tregs in the TME and the unmet clinical need to modulate Treg activity, the development of drugs targeting Tregs for cancer therapy has been slow.^9^^,^^10^ The overlapping intracellular signaling pathways of human Tregs and other T cells pose a major hurdle in the selective modulation of Treg function.^11^ Pre-clinical studies on monoclonal antibodies targeting surface markers such as CD25^12^^,^^13^ or CTLA4^14^ revealed efficiency in restoring anti-tumor immunity by depletion of Tregs. However, adverse effects may occur due to the overlapping expression of these proteins on activated T cells.

The transcription factor FoxP3 is a linage-defining molecule, the expression of which reprograms T cell development to Tregs.^15^ FoxP3 is upregulated upon peripheral induction and reprogramming of naive CD4^+^ T cells, yielding immunosuppressive Tregs.^16^ FoxP3 is a key transcription factor regulating the expression of several genes required for important Treg functions, such as CD25, CTLA4, CD73, and CD39, suggesting it as a potential target for cancer immunotherapy. So far, small molecules that indirectly affect FoxP3 upregulation or activation to turn off Tregs, for example, MEK inhibitors,^17^ PI3Kδ inhibitors,^18^^,^^19^ and the small molecule NLOC-015A that targets EGFR/MAP2K1/mTOR/YAP1^20^ have been proven to boost anti-tumor immune responses *in vitro* but have not progressed to *in vivo* validation due to low selectivity versus Teffs. A few studies have reported on the development of macromolecule regulators directly acting on FoxP3, including the FoxP3 antisense oligonucleotides (ASOs) AZD-8701 from AstraZeneca,^21^ a p60 peptide targeting FoxP3 degradation,^22^^,^^23^^,^^24^ and Foxp3-#32, which is a T cell receptor (TCR) mimicking antibody specific for FoxP3 epitopes presented on HLA-A∗02:01.^25^ However, diffusible small molecules with effects on FoxP3 regulation would still be in demand due to their availability to intra-tumoral lymphocytes.

Here, we established a flow-cytometry-based assay for phenotypic high-throughput screening using T cells from healthy human donors to screen for small molecule regulators of FoxP3. From screening of a drug repurposing library, we identified the anti-malaria drug quinacrine dihydrochloride dehydrate (QDD) as one promising candidate for reducing FoxP3 level in Tregs. After several rounds of *in silico* searches and validation, we defined a set of 9-amino acridine derivatives that control FoxP3 downregulation. Cell-based experiments showed that these acridines have significant effects on Treg suppressive functions toward Teffs. The compounds also inhibited the expression of Treg-specific markers and selectively regulated Treg signaling pathways, possibly interfering with FoxP3 DNA binding activity and through an auto-regulatory loop inhibiting FoxP3 expression. Treatment of cancer patient samples *ex vivo* and *in vivo* experiments in a mouse tumor model revealed anti-tumor immune regulatory properties of these compounds.

## Results

### Screening of a drug repurposing library identified quinacrine as a regulator of FoxP3

**Figure 1:**
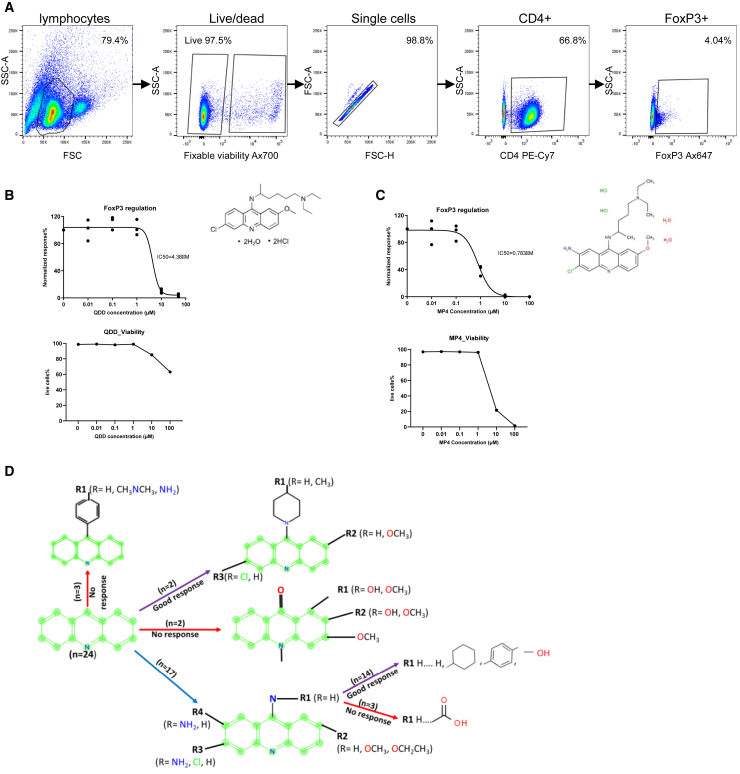
Validation of quinacrine and quinacrine-like acridines that downregulate FoxP3 expression in human T cells (A–C) CD3^+^ T cells were treated with drugs for 24 h, then stained with CD4 and FoxP3 antibodies for flow cytometry analysis. (A) FACS plots shows the representative gating strategy. Concentration-response curves for QDD (B) and MP4 (C) for downregulation of FoxP3 are shown, as well as live cells (%) at tested concentrations. Normalized response: FoxP3% in CD4^+^ T cells normalized to mock treatment (*n* = 3 donors). Viability data are presented as mean ± SEM. (D) Overview of structure-activity relation (SAR) for 9-amino-acridine structures. The acridine backbone is set as the starting point in the illustration (left). The purple lines represent compounds with potent ability to downregulate FoxP3, while the red lines represent compounds with no or poor ability to regulate FoxP3. The blue arrow represents the group of compounds with the replacement of H in the 9-N-R1 position from the backbone. *n* = number of compounds in each group.

To search for potential small molecular FoxP3 inhibitors, we screened the Prestwick library of approved drugs (1,522 compounds) for compounds that regulated the expression of FoxP3 using a novel high-throughput flow cytometry protocol. Primary CD3^+^ T cells from healthy donors were treated with compounds from the Prestwick library at 10 μM for 16 h, followed by a high-throughput flow cytometry analysis measuring FoxP3% in CD4^+^ T cells (for workflow, see Figure S1A). Compounds that reduced FoxP3 levels compared to DMSO were selected for further validation. The first verified hit was quinacrine dihydrochloride dihydrate (QDD), where we observed a concentration-dependent FoxP3 downregulation with an IC_50_ of less than 5 μM in all donors tested (Figures 1A and 1B; Table 1, group 0; Figure S2A). QDD was also confirmed to downregulate FoxP3 in Tregs by gating out CD127^+^ cells in both non-stimulated and stimulated T cells (Figure S1B).

**Table 1:** List of compounds validated in the screen

Compound group	Compound	Chemical name (IUPAC traditional)	IC50 on FoxP3 downregulation (μM)	Maximal suppressive effect	Compound structure
Group 0	Quinacrine dihydrochloride dihydrate	6-chloro-N-[5-(diethylamino)pentan-2-yl]-2-methoxyacridin-9-amine dihydrate dihydrochloride	4.38	95.9%	
Group 0	Amiloride	3,5-diamino-6-chloro-N-(diaminomethylidene)pyrazine-2-carboxamide	N/A	8.5%	
Group 0	Triamterene	6-phenylpteridine-2,4,7-triamine	N/A	18.8%	
Group 0	Carbamazepine	benzo[b][1]benzazepine-11-carboxamide	N/A	6.5%	
Group 0	Dipyridamole	2-[[2-[bis(2-hydroxyethyl)amino]-4,8-di(piperidin-1-yl)pyrimido[5,4-*d*ay]pyrimidin-6-yl]-(2-hydroxyethyl)amino]ethanol	N/A	1.7%	
Group 1	Amodiaquine	4-[(7-chloroquinolin-4-yl)amino]-2-[(diethylamino)methyl]phenol dihydrate dihydrochloride	16.7	55.1%	
Group 1	Primaquine	N4-(6-methoxyquinolin-8-yl)pentane-1,4-diamine; bis(phosphoric acid)	>100	16.4%	
Group 1	Chloroquine	7-chloro-N-[5-(diethylamino)pentan-2-yl]quinolin-4-amine; bis(phosphoric acid)	50.3	49.7%	
Group 1	Hydroxychloroquine	hydroxychloroquine; sulfuric acid (2-[4-[(7-chloroquinolin-4-yl)amino]pentyl-ethylamino]ethanol;sulfuric acid)	60.5	58.9%	
Group 2	MP2	acridin-9-amine hydrochloride	4.742	98.8%	
Group 2	MP3	6-chloro-2-methoxy-9-(4-methylpiperidin-1-yl)acridine	5.259	92.8%	
Group 2	MP4	3-chloro-N9-[5-(diethylamino)pentan-2-yl]-7-methoxyacridine-2,9-diamine	0.783	100%	
Group 2	MP5	9-(piperidin-1-yl)acridine	0.86	100%	
Group 3	MP7	Amsacrine (*N*-[4-(acridin-9-ylamino)-3-methoxyphenyl]methanesulfonamide)	8.8	73.7%	
Group 3	MP8	2-hydroxy-1,3-dimethoxy-10-methyl-9,10-dihydroacridin-9-one	111	39.4%	
Group 3	MP9	ethacridine lactate (7-ethoxyacridine-3,9-diamine;2-hydroxypropanoic acid)	7.5	100%	
Group 3	MP10	arborinine (1-hydroxy-2,3-dimethoxy-10-methylacridin-9-one)	>100	13.8%	
Group 3	MP11	9-Amino-6-chloro-2-methoxyacridine	1.3	94.5%	
Group 3	MP12	N1-(6-chloro-2-methoxyacridin-9-yl)benzene-1,4-diamine	8	79.9%	
Group 3	MP13	N4-(acridin-9-yl)-N1,N1-dimethylbenzene-1,4-diamine	5.8	92.0%	
Group 3	MP14	N-(4-methylphenyl)-9,10-dihydroacridin-9-imine	3.9	96.4%	
Group 3	MP15	4-({7-chloro-2-methoxybenzo[b]1,5-naphthyridin-10-yl}amino)-2,6-bis[(pyrrolidin-1-yl)methyl]phenol	6.6	97.1%	
Group 4	MP16	9-phenylacridine	No response	12.4%	
Group 4	MP17	3-[(acridin-9-yl)amino]propan-1-ol	1.93	94.6%	
Group 4	MP18	N-cyclohexylacridin-9-amine	1.13	94.8%	
Group 4	MP19	3-[(acridin-9-yl)amino]propanoic acid	No response	25.4%	
Group 4	MP20	2-[(acridin-9-yl)amino]pentanedioic acid	Bad response	32.5%	
Group 4	MP21	4-(acridin-9-ylamino)butanoic acid	No response	2.6%	
Group 4	MP22	4-(acridin-9-yl)-N,N-dimethylaniline	No response	14.8%	
Group 4	MP23	4-(acridin-9-yl)aniline	Bad response	34.1%	
Group 4	MP24	N-[4-(4-methylpiperazin-1-yl)phenyl]acridin-9-amine dihydrochloride	0.99	93.4%	
Group 4	MP25	(2S)-2-(acridin-9-ylamino)pentanedioic acid	Bad response	44.0%	

All compounds listed in the table were tested in CD3^+^ T cells from healthy donors at concentrations of 0-0.01-0.1-1-10-100 μM for 24 h, followed by flow cytometry staining for FoxP3 and CD4. The IC50 of each compound was determined in GraphPad Prism by fitting the response curve after normalization of FoxP3% in CD4 in the drug-treated group to DMSO control (set DMSO control = 100%). Maximal suppressive effect assessed as maximal change of FoxP3% normalized to DMSO at the concentrations tested (0%–100%). (*n* = 3 donors.)

### Quinacrine-related structures demonstrate diverse effects on FoxP3 levels

QDD is a Food and Drug Administration (FDA)- and European Medicines Agency (EMA)-approved anti-malaria drug in use since 1932 and later repositioned for anti-helminthic and anti-rheumatic use.^26^ Since then, several drugs with related, yet different structures have been developed for anti-malaria treatment. Therefore, four of these other anti-malaria drugs belonging to the aminoquinoline group, amodiaquine, primaquine, chloroquine, and hydroxychloroquine, were tested. However, they were found to be less potent in their regulatory effect on FoxP3 levels than QDD (Table 1, group 1; Figure S2B).

*In-silico* prediction based on the structure of QDD, which belongs to the group of 9-amino-acridines, was applied to identify more effective compounds. In the first round, compounds MP2, MP3, MP4, and MP5 were identified to be working efficiently in downregulating FoxP3 levels (Table 1, group 2; Table S1; Figure S2C). Interestingly, compounds MP4 and MP5 had significantly lower IC50 ˂1 μM, indicating more than 5-fold higher efficacy in FoxP3 downregulation than QDD, (Figures 1C; Table 1; Figure S2C). Analysis of the SAR of compounds in group 2 identified an acridine ring, which could be essential for suppressing FoxP3 expression. Therefore, a small library of similar acridine and acridinone compounds (Table 1, group 3 and 4; Table S1; Figures S2D and S2E) was assembled by *in-silico* prediction and assessed for their effects on FoxP3 regulation. As summarized in Table 1, most of the acridine compounds in groups 3 and 4 showed promising effects on FoxP3 downregulation with IC50 ranging from 1 μM to 10 μM. However, acridinones MP8 and MP10 and some compounds from group 4 did not affect FoxP3 regulation or came out with IC50 above 100 μM. Our data support the notion that a set of QDD-derived 9-amino-acridines with specific structural determinants are critical for FoxP3 downregulation (Figure 1D).

### Blockade of Treg suppressive functions by QDD and its 9-amino-acridine analogs

**Figure 2:**
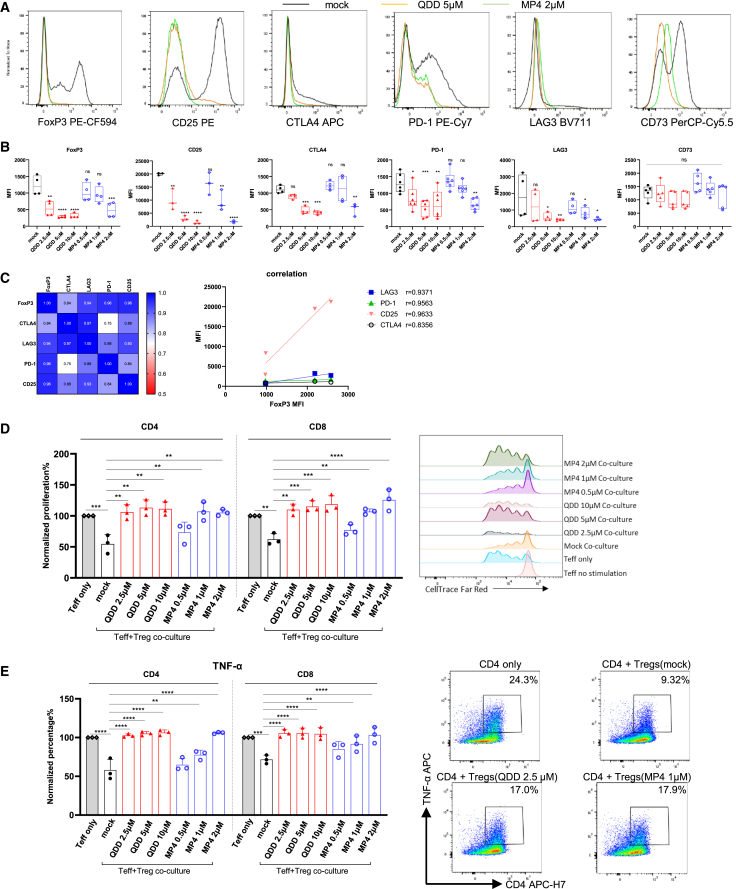
QDD and MP4 impair the suppressive functions of Tregs toward Teff cells Isolated CD4^+^CD25^+^CD127^dim/-^ Tregs from healthy donor buffy coats were stimulated with CD2/CD3/CD28 beads while treating with QDD/MP4 at specified concentrations for 48 h and subjected to the indicated analyses. (A–C) Expression of FoxP3, CTLA-4, LAG3, CD25, PD-1, and CD73 in Tregs were measured by flow cytometry. (A) Representative histograms show expression level of each protein with or without compound treatment. (B) The expression level of each marker was represented as mean fluorescence intensity (MFI) on the y axis. Boxplots show all data points with median and min to max (*n* = 3 donors). (C) Correlation of expression of Treg markers is presented as a heatmap (left), and a plot with FoxP3 MFI (x axis) vs. MFI of other markers (right). The Pearson correlation coefficient, r, is calculated and the plots are generated in GraphPad Prism. (D) After 48 h of pre-activation and compound treatment, Tregs were co-cultured with CellTrace Far Red-stained effector CD4 or CD8 Teff at 1:2 ratio for 96 h. Proliferation of effector CD4 or CD8 Teffs was analyzed by flow cytometry and compared to the proliferation of Teffs alone, which is set to 100%. The FACS histograms on the right show the proliferating Treff assessed by CellTrace Far Red signal. (E) Production of TNFα in CD4/CD8 Teff cells was determined by intracellular flow cytometry staining after co-culture with Tregs and is presented as the percentage of TNFα producing cells normalized to Teff only (set to 100%). FACS plots on the right shown representative gating of TNFα in CD4 Teff. Data are represented as mean ± SEM (*n* = 3–5 donors). *p* value was determined by ordinary one-way ANOVA (B) or two-way ANOVA (D and E). ∗*p* ˂ 0.05, ∗∗*p* ˂ 0.01, ∗∗∗*p* ˂ 0.001, ∗∗∗∗*p* ˂ 0.00001. ns, not significant.

Having defined a set of 9-amino-acridine compounds that could efficiently reduce FoxP3 expression in CD4^+^ T cells using our cell-based assay, we next wanted to test if the alterations of FoxP3 levels by these compounds could affect Treg functions. QDD and the 5-fold more efficient and water-soluble compound MP4 were then included in functional assays using concentrations according to their individual IC_50_ with respect to the regulation of FoxP3 levels. Isolated CD4^+^CD25^+^CD127^dim/-^ Tregs from healthy donors treated with QDD or MP4 revealed significantly reduced expression of surface markers such as CD25, CTLA-4, LAG3, and PD-1, consistent with the downregulation of intracellular FoxP3 at almost all concentrations tested (Figures 2A and 2B; Figure S3A). In addition, the FoxP3 expression level was confirmed to be positively correlated with other Treg markers as shown in Figure 2C. Next, we examined the effects of QDD and MP4 on the immune suppressive functions of Tregs, using our established *in vitro* Treg suppression assay for compound screening.^18^ Pre-activated Tregs could suppress the proliferation of both effector CD4 and CD8 T cells in co-culture (Figure 2D, mock in co-culture vs. Teff only; Figure S3B). After QDD or MP4 treatment during pre-activation, Treg suppressive function toward Teffs was dramatically reduced, represented as rescued proliferation of Teffs in co-culture with compound-treated Tregs to levels as for Teffs only (Figure 2D, compound-treated vs. mock in co-culture). QDD completely inhibited the suppressive capacity of Tregs at all concentrations tested from 2.5 μM to 10 μM, while MP4 was equally efficient at 1–2 μM, consistent with their observed efficacy in regulating FoxP3. In addition, the production of tumor necrosis factor alpha (TNFα) in CD4^+^ and CD8^+^ Teff cells was recovered in co-culture with QDD- or MP4-treated Tregs (Figure 2E). However, in QDD-treated Treg cultures there was significant reduction of viable cells after two days at higher concentrations (Figure S3A), implying the recovery of Teff proliferation in co-culture might be due to the toxicity of QDD in Tregs (low percentage of Tregs after co-culture shown in Figure S3B). For this reason, we also performed Treg suppression experiments with a protocol where we recounted Tregs after pre-treatment to ensure a Treg:Teff ratio at 1:2 in the co-cultures (Figure S3C). In these experiments, Tregs pre-treated with QDD at 2.5 μM and with MP4 at all concentrations were preserved after co-culture, while the toxicity of QDD at high concentrations resulted in loss of Tregs in co-culture even after QDD was removed (Figure S3C). Importantly, the Treg suppressive functions on Teff were significantly reduced by QDD (2.5 μM) and MP4 (2 μM) pretreatment (Figure S3D), represented as recovery of Teff proliferation. In summary, we could observe a significant blockade of Treg marker expression and Treg suppressive function toward Teffs by QDD and its 9-amino-acridine analog MP4 at non-toxic concentrations, indicating a FoxP3-dependent mechanism of action among these similar compounds.

### QDD and its 9-amino-acridine analogs selectively work on Tregs rather than Teffs

**Figure 3:**
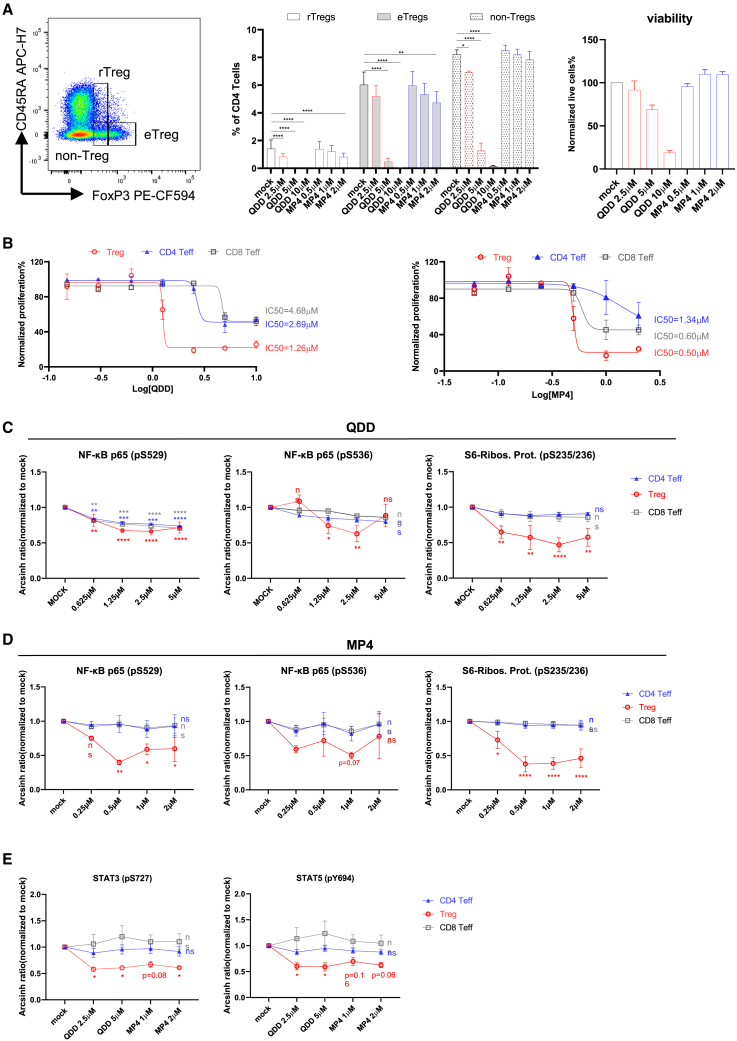
Selective inhibitory effects of QDD and MP4 on Tregs over Teffs (A) CD3^+^ T cells from healthy donors were treated with compounds for two days under TCR stimulation. Different Treg populations were determined by gating for CD45RA versus FoxP3 in CD4 T cells (left FACS plot) by flow cytometry. FoxP3 positive cells (%) in each Treg population (middle) and cell viability (right) are shown. (B) Purified CD4 Teff, CD8 Teff, and Tregs from healthy donor CD3^+^ T cells were stained with CellTrace Far Red and treated with QDD or MP4 at indicated concentrations for 4 days, under stimulation by CD2/CD3/CD28 beads. Cell proliferation was measured by flow cytometry for CellTrace Far Red-positive cells and normalized to that of mock-treated cells. IC_50_ was calculated in each population. (C–E) Representative phosphorylation signals were measured by phospho-flow cytometry in healthy donor CD3^+^ T cells that were treated with QDD or MP4 for 30 min at specified concentrations, by gating on CD8 Teff (CD8^+^), CD4 Teff (CD4^+^CD25^−^FoxP3^-^) and Treg (CD4^+^CD25^+^FoxP3^+^) populations. Phospho-flow staining was performed right after 30 min treatment of compounds (C and D) or following IL-2 stimulation for 45 min (E). MFI of each protein phosphorylation level is represented as the arcsin ratio normalized to the mock-treated group. *n* = 3 healthy donors. Mean ± SEM is shown, and statistics represent calculation of compound groups against mock in each population. ∗*p* ˂0 .05, ∗∗*p* ˂ 0.01, ∗∗∗*p* ˂ 0.001, ∗∗∗∗*p* ˂ 0.00001; two-way ANOVA.

To assess the specificity of 9-amino-acridine analogs, we first investigated their effects on different Treg populations identified by combined staining for CD45RA and FoxP3.^16^^,^^27^^,^^28^ Interestingly, MP4 reduced the fraction of eTregs (CD45RA^−^FoxP3^high^) and rTregs (CD45RA^+^FoxP3^low^) more potently than non-Tregs (CD45RA^−^FoxP3^low^) in stimulated T cells (Figure 3A). However, QDD downregulated all Treg populations, which appeared to be due to toxicity, particularly at higher concentrations (Figure 3A). In non-stimulated T cells, QDD and MP4 both had effects in the rTregs population (Figure S4A). Next, we examined the effect of QDD and MP4 on the proliferation of different T cell subsets. After 4 days of culture in the presence of QDD or MP4, the proliferation of all purified cell types was inhibited at the higher concentrations tested (Figure 3B). However, Tregs were more sensitive to the QDD or MP4 treatment than Teffs, with the lowest IC_50_ of QDD or MP4 on proliferation inhibition, implying a selectivity toward Tregs. Notably, at most of the concentrations tested, cell death of CD4^+^ and CD8^+^ Teffs and Tregs was not significantly increased, except at the highest concentrations used (Figures S4B and S4C), suggesting that these compounds impact proliferation and Tregs functions rather than displaying a cytotoxic effect. When tested in mixed T cells, MP4 inhibited proliferation of Tregs somewhat more than that of Teffs and more potently than QDD (Figure S4D). In addition, MP4 only affected CD25 expression in Tregs (Figure 2B) but not in Teffs (Figure S4E). In contrast, QDD exhibited downregulation of CD25 in all cell types, which might be due to high toxicity. These data suggest a more selective and tolerable effects of MP4 in Tregs than that of the original hit QDD.

During the past decades, anti-cancer effects of quinacrine have been tested on some types of tumor cells. Quinacrine was reported to induce apoptosis and suppress tumor cell proliferation through signaling pathways such as p53/nuclear factor κB (NF-kB),^29^ wnt/β-catenin^30^ and AKT/mTOR.^31^ Therefore, we evaluated whether the selectivity of 9-amino-acridines on Treg proliferation is due to differential effects in regulating tonic T cell signaling by phospho-flow assays in resting primary T cells. We observed inhibition of phosphorylation of NF-kB Ser529 and Ser536 by QDD in a concentration-dependent manner but did not observe differences between Tregs and Teffs (Figure 3C). Interestingly, MP4 revealed significant inhibition of NF-kB phosphorylation in Tregs compared to Teffs, suggesting a more potent and selective function of MP4 than QDD on NF-kB signaling in Tregs (Figure 3D). By examining other protein phosphorylation events in TCR signaling pathways, we found that phosphorylation of the S6 ribosomal protein (S6-Rp) was dramatically and selectively reduced in unstimulated Tregs upon QDD and MP4 treatment (Figures 3C and 3D), implying possible regulation of the mTOR pathway that is important for Treg activation. MP4 also selectively inhibited S6-Rp phosphorylation and other TCR signaling events, such as CD3 and AKT/PKB in Tregs both in the absence and presence of TCR activation (Figure S5).

The IL-2 signaling pathway is dominant in Tregs due to the abundant expression of the interleukin-2 receptor alpha chain (IL2RA, CD25) and regulates Treg suppressive functions through STAT3 and STAT5 signaling cascades.^32^ We observed selective inhibition by QDD and MP4 of STAT3 (pS727) and STAT5 (pY694) phosphorylation in Tregs rather than Teffs under IL-2 stimulation (Figure 3E).

Taken together, the selective inhibitory functions of QDD and MP4 on Treg proliferation and signaling suggest a distinct regulatory mechanism in FoxP3^+^ Tregs.

### Direct binding of 9-amino-acridines to FoxP3 interferes with FoxP3-DNA interaction and inhibits FoxP3-dependent gene regulation

To explore how 9-amino acridines downregulate FoxP3, directly or indirectly, a low-molecular-weight compound screening (low molecular weight [LMW] screen) was performed using surface plasmon resonance (SPR) analysis to determine affinities between FoxP3 and all the compounds listed in Table 1. Interestingly, almost all the 9-amino-acridine analogs that displayed a strong ability to reduce FoxP3 levels in the cell-based assay revealed an affinity for the FoxP3 protein, such as QDD, MP4, and MP24 (all tested at 50 μM) (Figure S6A). In contrast, we observed low or no affinity for FoxP3 with compounds that had little or no effects on FoxP3 downregulation (such as MP20 and MP25). Furthermore, we confirmed the concentration-dependent binding of QDD and MP4 to immobilized FoxP3 protein (Figure S6B) with dissociation constant (K_D_) values corresponding to the observed IC_50_ in the cell-based assay, implying that the function of 9-amino-acridine analogs on FoxP3 regulation might be due to their direct binding ability.

**Figure 4:**
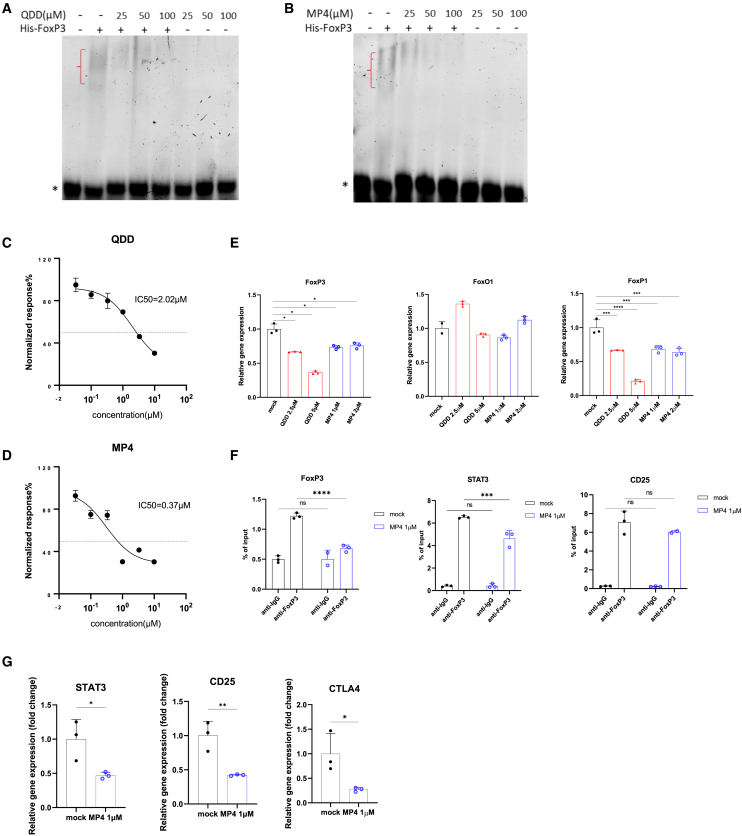
Quinacrine and MP4 interfere with the DNA binding activity of FoxP3 (A and B) EMSAs were performed to assess FoxP3 binding to DNA *in vitro*. QDD (A) or MP4 (B) at different concentrations were pre-incubated with His-FoxP3-ΔN for 30 min before introducing a double-stranded DNA sequence to allow binding. Bands marked by red brackets “{” represent the FoxP3-DNA complex, which is retarded in the gel compared to the mobility of free DNA denoted by “∗”. (C and D) AlphaScreen assays determined the ability of QDD (C) or MP4 (D) to compete DNA binding to FoxP3 *in vitro*. The signal from His-FoxP3 interacting with biotinylated-DNA was normalized to that from the counter screen. (E) qPCR was performed with total RNA isolated from CD3^+^ T cells treated with QDD or MP4 at indicated concentrations for 24 h. Relative mRNA expression levels of *FoxP3*, *FoxP1*, and *FoxO1* were normalized against *RPS9* internal control. (F) The binding of FoxP3 to *FoxP3*, *STAT3* and *CD25* gene promoter regions was measured by ChIP-qPCR in total chromatin DNA isolated from Tregs that were treated with MP4 for 4 h. Relative binding levels were normalized to input control (1% of total chromatin DNA). (G) RNA from Tregs treated with MP4 for 4 h were analyzed by qPCR to measure the gene expression levels of *STAT3*, *CD25*, and *CTLA4*, normalized to *RPS9* internal control. Mean ± SEM (*n* = 3) is shown in the graphics. One-way ANOVA (E), two-way ANOVA (F), and unpaired t test (G) were used to determine *p* values. ∗*p* ˂ 0.05, ∗∗*p* ˂ 0.01, ∗∗∗*p* ˂ 0.001, ∗∗∗∗*p* ˂ 0.00001. ns, no significant.

Some acridine compounds are reported to intercalate with DNA and inhibit topoisomerase, thereby affecting DNA replication and transcription. To examine whether the identified 9-amino-acridines could affect the DNA-binding activity of FoxP3, we performed electrophoretic mobility shift assays (EMSAs) in native gels to measure FoxP3 binding to DNA *in vitro*. As shown in Figures 4A and 4B, QDD and MP4 interfered with FoxP3-DNA binding in a concentration-dependent manner. Next, using an AlphaScreen-based assay for measuring FoxP3-DNA interaction, we observed remarkable inhibition of FoxP3-DNA binding by QDD and MP4, with IC_50_ of 2 μM and 0.37 μM, respectively, again revealing effects at similar concentrations as in the cell-based assays (Figures 4C and 4D). These data support our hypothesis that 9-amino-acridines act on FoxP3 downregulation through direct interference with FoxP3-DNA binding activity. It is also important to note that there is a FoxP3 auto-regulatory loop in Tregs where FoxP3 regulates its own expression.^33^ Interestingly, the levels of *FoxP1* mRNA by qPCR analysis were also reduced in a concentration-dependent manner in parallel with *FoxP3* mRNA (Figure 4E), which might be due to the required dimerization of FoxP3 and FoxP1 in the auto-regulatory loop.^34^ Importantly, the expression of another Forkhead protein *FoxO1* was not affected by QDD and MP4, indicating that 9-amino-acridines target preferentially FoxP proteins (Figure 4E) and not other Forkhead proteins or DNA generally.

To further investigate the effects of quinacrine-like acridines on FoxP3-DNA interaction and the downstream gene regulation, FoxP3 chromatin immunoprecipitation (ChIP) followed by qPCR was performed in Tregs treated with MP4. QDD had strong unspecific binding effects in IgG control samples and was omitted from the ChIP-qPCR analysis (data now shown). The binding of the FoxP3 protein to the promoter regions of *FoxP3* and *STAT3* was significantly reduced in MP4-treated Tregs, compared to the mock group (Figure 4F). Even though FoxP3 binding to the *CD25* promoter did not show a significant difference between mock and MP4 treatment, RT-PCR results from the ChIP samples displayed downregulation of *CD25* gene expression, in addition to *STAT3* and *CTLA4* under MP4 treatment (Figure 4G). Taken together, these data suggest that 9-amino-acridines primarily work by inhibition of FoxP3-DNA binding activity, thus interfering with FoxP3-dependent gene regulation.

### Quinacrine-like acridines boost immune response in cancer patient samples

**Figure 5:**
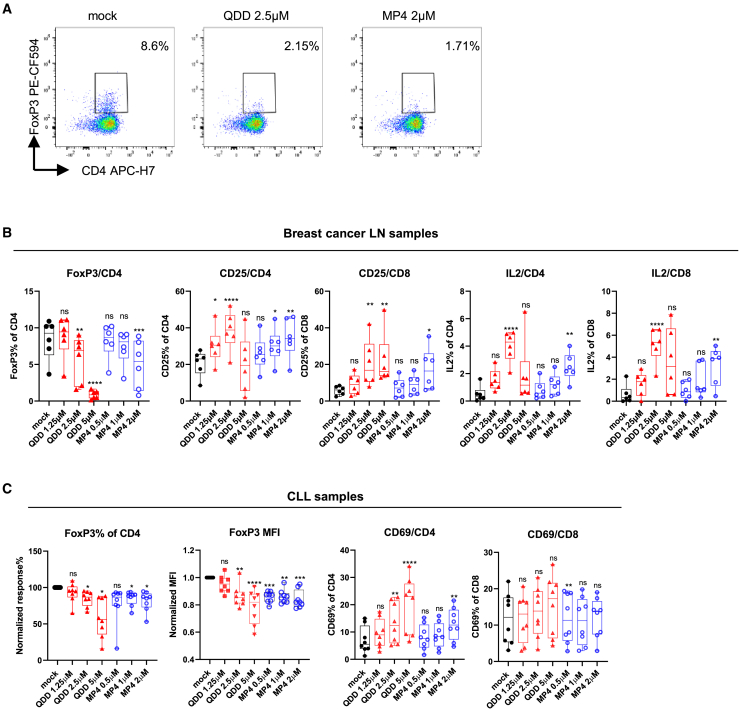
Effects of QDD and MP4 on boosting T cell activation in cancer patient samples (A and B) Cells isolated from breast cancer patient lymph nodes (LNs) were treated with QDD or MP4 at specified concentrations for 48 h. BFA was added to the culture 6 h before harvest. (A) The representative gating strategy of FoxP3 in CD4^+^ T cells. (B) Expression of activation marker CD25 and intracellular IL-2 in CD4^+^ or CD8^+^ T cells were determined by flow cytometry analysis (*n* = 6 patients). (C) PBMCs isolated from CLL patients’ blood were treated with QDD or MP4 for 48 h and FoxP3 and CD69 expression measured in CD4^+^ or CD8^+^ T cells by flow cytometry (*n* = 8 patients). Normalized responses: FoxP3% in CD4^+^ T cells was normalized to DMSO control and FoxP3 MFI normalized to DMSO control. Boxplots show all data points with median and Min to Max. Significant differences between mock and compound-treated groups were determined by two-way ANOVA. ∗*p* ˂ 0.05, ∗∗*p* ˂ 0.01, ∗∗∗, *p* ˂ 0.001, ∗∗∗∗*p* ˂ 0.00001. ns, not significant.

We next evaluated the possible application of the 9-amino-acridines in promoting anti-tumor immune responses by restoring Teff activity. First, we examined the effects of 9-amino-acridines on axillary lymph node (LN) samples collected from breast cancer patients. FoxP3 levels were downregulated in patient samples after QDD and MP4 treatment for 48 h, especially at high concentrations (QDD at 2.5 μM, MP4 at 2 μM) (Figures 5A and 5B). In addition, QDD and MP4 treatment boosted intrinsic activation of Teffs, as evident from their CD25 expression and increased production of cytokines such as IL-2, interferon (IFN)γ, and TNFα (Figures 5B; Figures S7A and S7B), which implies a functional recovery. Notably, the concentration of MP4 (2 μM) effective in FoxP3 downregulation did not introduce significant cell death, compared to effective concentrations of QDD that also introduced toxicity at both 2.5 μM and 5 μM (Figure S7C). Next, QDD and MP4 were tested on peripheral blood mononuclear cells (PBMCs) from chronic lymphocytic leukemia (CLL) patients, where FoxP3 downregulation in Tregs (normalized response) was accompanied by a stronger intrinsic activation of Teffs (higher CD69% in CD4 or CD8; Figure 5C). In summary, these results from cancer patients’ immune cells examined *ex vivo* indicated that 9-amino-acridines might boost anti-tumor immune activity through blockade of Treg suppressive functions.

### Anti-tumor immune regulation properties of quinacrine-like acridines *in vivo*

**Figure 6:**
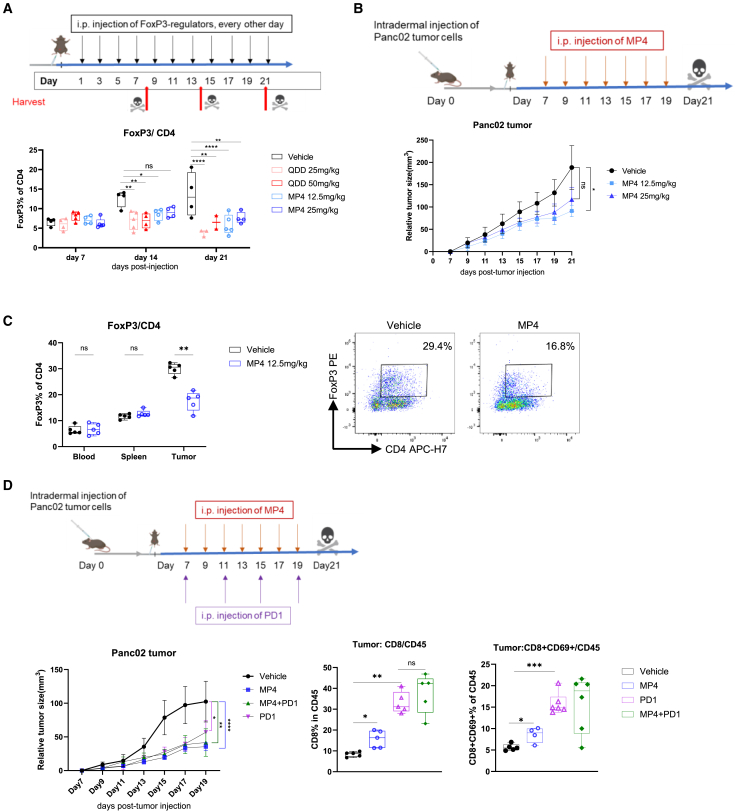
*In vivo* effects of QDD-like acridines in a mouse tumor model (A) Titration of QDD and MP4 in C57BL/6 mice healthy mice by intraperitoneal injections is illustrated in the upper figure. Blood samples collected at each specified time point were measured for FoxP3% in CD4^+^ T cells by flow cytometry. (B and C) Panc02 tumor-bearing C57BL/6 mice were administered Kolliphor HS15-formulated-MP4 or vehicle for 14 days, from 7 days after tumor cell injection. (B) The treatment scheme is illustrated in the upper figure, while the tumor growth curve below shows the relative tumor size of each time point normalized to that of day 7 in each group. (C) FoxP3% in CD4 in blood, spleen, and tumor samples were measured by flow cytometry (left), illustrating FoxP3 gating in the pseudo color plots (right). (D) Mice bearing Panc02 tumors were treated with vehicle, MP4 or PD1 single treatment, and MP4+PD1 treatment following the illustrated scheme. Proportions of tumor infiltrating CD8^+^ T cells and activated CD8^+^T cells (CD8^+^CD69^+^) in CD45^+^ cells were determined by flow cytometry. *n* = 5–6 mice per group. Boxplots show all data points with median and min to max. Two-way ANOVA (A–D, left) and one-way ANOVA (D, right) were used to determine *p* values. ∗*p* ˂ 0.05, ∗∗*p* ˂ 0.01,∗∗∗, *p* ˂ 0.001, ∗∗∗∗*p* ˂ 0.00001. ns, no significant.

To evaluate the effects of quinacrine-like acridines *in vivo*, their effect on FoxP3 was first verified by treating mouse splenocytes isolated from C57BL/6 mice (Figures S8A and S8B), demonstrating similar effects as in human primary T cells. To determine the optimal treatment dose and scheme, QDD or MP4 formulated in Kolliphor HS15 were next administered to healthy mice by intraperitoneal injections every other day, and blood and splenocytes were harvested for flow cytometry analysis at different time points as illustrated in Figure 6A. FoxP3 levels were reduced to around 50% in blood samples after 2 and 3 weeks of treatment in all QDD and MP4 groups (Figure 6A). However, considering the mortality at high doses of QDD (50 mg/kg) observed from day 14 (Figure S8C), as well as the potential general toxicity after three weeks of treatment (represented as lower lymphocyte count in Figure S8D), we decided to examine the effects only of MP4 in mice tumor models for up to 2 weeks of administration. As illustrated in Figure 6B, MP4 inhibits tumor growth in a well-established syngeneic pancreatic cancer tumor model (Panc02) in C57BL/6 mice. Interestingly, MP4 at 12.5 mg/kg had a stronger inhibitory effect on tumor growth than at 25 mg/kg (Figure 6B), without introducing toxicity. One possible explanation can be that MP4 partially inhibited Teffs at a high dosage of 25 mg/kg. In the group receiving 12.5 mg/kg of MP4, Treg populations (CD4^+^FoxP3^+^) were reduced by 50% in tumor samples (Figure 6C), but not in blood and spleen, indicating a more specific targeting of Tregs in the TME contributing to the anti-tumor immune response. Importantly, we did not observe changes in tumor growth in nude mice bearing Panc02 tumors that were treated with MP4 or vehicle with the same scheme as C57BL/6 mice (Figure S8E), indicating an immune-related anti-tumor regulation by MP4.

Treatment with immune checkpoint inhibitors (ICIs) such as anti-PD1, have well-documented anti-tumor effects in humans^35^ and have also been shown to work in the Panc02 mouse tumor model.^36^^,^^37^ We tested the combined effects of MP4 and anti-PD1 therapy using the same Panc02 model. Mice from all groups survived after 21 days from tumor injection. As shown in Figure 6D, MP4 or PD1 single treatment inhibited tumor growth in a similar pattern, with enhanced infiltration and activation (CD69^+^) of CD8 T cells into the TME. However, we did not observe any significant synergistic effect of the combination of MP4 and anti-PD1 treatment.

## Discussion

Here, we report on establishing a novel high-throughput, flow-cytometry-based, phenotypic assay to screen for regulators of FoxP3 in human primary CD3^+^ T cells from healthy blood donors. By screening a drug repurposing library containing more than 1,500 approved drugs, we identified only a few candidates that downregulate FoxP3 from a hit list of 38 compounds. The low hit rate (0.3%) may be due to the low expression level of endogenous FoxP3 in primary T cells and the difficulty of verifying further reduced FoxP3 levels in T cells. To identify more hits in further screens of other libraries, prolonged stimulation of primary T cells by CD3 and CD28 to obtain *in vitro*-induced adaptive Tregs with higher FoxP3 levels could be considered.^38^ However, here we took the opportunity to characterize one of the more potent hits and a sub-library of analogs, which also serves to validate the method and targeting strategy.

We have characterized a family of 9-amino-acridines that can directly bind to FoxP3 or affect FoxP3-DNA binding activity and focused on their target specificities to avoid adverse effects. Since FoxP3 belongs to the Forkhead class of transcription factors that have members essential for both tumor and normal cell proliferation, our findings that QDD and MP4 bind to FoxP3 without showing any affinity for members of other Forkhead sub-classes such as FoxC2 and FoxD1 (SPR data now shown) support the specificity of these compounds. Nevertheless, other drugs that work on signaling pathways required for FoxP3 regulation can also be good candidates, affecting the upstream FoxP3 gene regulation, the FoxP3-dependent auto-regulatory loop, or downstream signaling. Notably, FoxP1, which is an auto-regulatory loop regulator of FoxP3 gene regulation, was also reduced in QDD and MP4 treatment, suggesting the functional specificity of the compounds.

QDD has been evaluated for repurposing for tumor treatment beyond its original anti-malaria effects, showing induction of apoptosis through an NF-kB pathway.^39^ We also noticed the inhibition of mouse tumor cell proliferation by QDD *in vitro*. Interestingly, MP4 showed lower toxicity to mouse tumor cell lines, without affecting proliferation *in vitro* (data not shown). Indeed, MP4 displayed a more selective effect in Tregs through NF-kB and ribosome-S6 regulation, while little specificity of QDD was observed in NF-kB signaling regulation between Tregs and other T cell subsets.

Experimental depletion of Tregs using a FoxP3^DTR^ mouse model confirmed its effects on tumor growth inhibition with different types of cancer cell lines, such as B16F10 (melanoma),^40^ CT26 (colon cancer),^41^ and LLC1 (Lewis lung carcinoma).^42^ However, this could cause lethal autoimmunity in mice.^43^ In our study, MP4 administration led to a reduction of FoxP3 to around 50% after 2 weeks of treatment in both healthy mice and mice bearing Panc02 tumors. Interestingly, we did not observe any induction of autoimmune responses in healthy mice treated with MP4 for up to 21 days. This MP4-induced FoxP3 reduction was associated with reduced tumor growth and increased levels of tumor-infiltrating CD8 cells, indicating that MP4 may reduce tumor immune evasion by Tregs. In contrast to our findings with the Panc02 model, we did not observe any effect of MP4 in a B16F10 melanoma tumor growth, which may be due to the more aggressive growth of these tumors or less Tregs infiltration. Notably, FoxP3 ASO did not show effects in tumors with low Treg infiltration.^21^ Despite the fact that high levels of Tregs infiltration in the TME are associated with poor prognosis in most types of cancer,^7^ controversial findings in patients with some cancer types, such as colorectal cancer, show that higher FoxP3 expression is associated with better outcomes.^35^^,^^44^ Furthermore, prolonged injection of QDD and MP4 in healthy mice for up to 3 weeks was characterized by lower lymphocyte counts that may represent autoimmunity. Thus, well-designed treatment schemes based on the therapeutic window of the compound to maximize effect and specificity and avoid autoimmunity and variable effects in different tumor types should be considered in further *in vivo* evaluation of Tregs targeting agents.

As an alternative cancer immunotherapeutic strategy, small molecules targeting FoxP3 in Tregs could achieve higher effects than biologicals and macromolecules due to their ability to cross the cell membrane, their easier penetration into the TME, and their possible oral availability. Among our hits, QDD and MP4 are water soluble and could easily be formulated in Kolliphor HS15 for intraperitoneal injections, implying possible suitability in clinical application. Other 9-amino-acridines that have similar effects as MP4, such as MP5, were also tested in our study. However, MP5 was only soluble in DMSO and could not be formulated in Kolliphor HS15. Therefore, further search of compounds based on the 9-amino-acridine structures, especially MP4, which has high water solubility, could help with the identification of druggable compounds for further *in vivo* clinical investigations. In addition, the upcoming possibilities to modify compounds with other small molecules, such as PROTACs, to specifically target FoxP3 degradation in tumor-infiltrating Tregs^24^^,^^36^ could be an alternative strategy to achieve higher selectivity and less toxicity. Furthermore, as PD-1/PD-L1 and CTLA4 inhibitors have been extensively evaluated clinically in patients with many cancer types, combinations of FoxP3 regulators and ICIs may be possible to test where ICIs do not work optimally. Even though our result did not show syngeneic effects of MP4 and anti-PD1 treatment in mice bearing Panc02 tumors, further tests of combinations with anti-CTLA4 or treatments targeting other tumor immune escape mechanisms such as PGE_2_/adenosine pathways could be further explored.

In summary, our data suggest that targeting FoxP3 through small molecules may be a therapeutic strategy to inhibit Tregs in the TME and enhance anti-tumor responses potently, implying potential application in patients as a stand-alone treatment or in combination with other therapies. MP4 may also be a candidate for further characterization and/or derivatization and as a tool compound to assess the level of Treg-mediated suppression for example, in patient samples *ex vivo*.

### Limitations of the study

We report on how a group of 9-amino-acridines downregulate FoxP3. We could, however, not exclude their toxic effects on both Tregs and Teffs over the range of tested concentrations. Moreover, the possible DNA intercalating properties of this series of compounds made it difficult to investigate their mechanism of action. Therefore, we do not argue that these compounds would have potential therapeutic application. However, their characterization serves to validate this screening approach; the compounds may have value as research tools and to assess the level of Treg-mediated tumor immune evasion in patient tumor samples and as a steppingstone for a further search for small molecule Treg inhibitors that may have future application as immunotherapy.

## Resource availability

### Lead contact

Further information and requests for reagents should be directed to and would be fulfilled by the lead contact, Kjetil Taskén (kjetil.tasken@medisin.uio.no).

### Materials availability

This study did not generate new unique reagents.

### Data and code availability

• All data reported in this paper will be shared by the lead contact upon reasonable requests.
• This paper does not report original code.
• Any additional information required to reanalyze the data reported in this paper is available from the lead contact upon request.

## Acknowledgments

We would like to thank Martine Schrøder and Mentowa Fürst Bright for expert technical assistance. We thank Alexandra Gade and Eirin Solberg at the High-Throughput Chemical Biology Screening Platform at Centre for Molecular Medicine Norway (NCMM), University of Oslo, for assistance with the high-throughput flow cytometry-based screen. We would also like to thank Selma Cornillot-Clément for help with the Prestwick library screen and Alexandra R. Dukic for the help with the Biacore SPR assay setup.

This work was supported by the Regional Health Authority for South-Eastern Norway grant 2020045 (K.T.) and grant 2019057 (H.E.G.R.), the Research Council of Norway grant 315538/H10 (K.T.), the Research Council of Norway grant 328827 (K.T. and H.E.G.R.) and 10.13039/100008730Norwegian Cancer Society grant 215850 (K.T.). The graphical abstract and the treatment schemes in Figure 6 were generated with BioRender (https://BioRender.com).

## Author contributions

Conceptualization, K.T., Q.W., and J.L.; methodology, Q.W., H.F., J.L., S.W., J.K., S.S.S., H.E.G.R., and R.A.; investigation, Q.W., H.F., J.L., S.W., I.H.R., R.A., and K.T.; resources, K.T. and H.E.G.R.; writing (original draft), Q.W. and K.T.; writing (review and editing), all authors.

## Declaration of interests

The authors declare no competing interests.

## STAR★Methods

### Key resources table

**Table:** 

REAGENT or RESOURCE	SOURCE	IDENTIFIER
**Antibodies**		

Mouse anti human CD4 PE-Cy7 (Clone SK3)	BD Biosciences	Cat# 557852; RRID: AB_396897
Mouse Anti-Human CD4 APC-H7 (RPA-T4)	BD Biosciences	Cat# 560158; RRID: AB_1645478
Mouse anti human CD8 BV786 (Clone RPA-T8)	BD Biosciences	Cat# 563823; RRID: AB_2687487
Mouse anti human CD3 PerCP-Cy5.5 (Clone UCHT1)	BD Biosciences	Cat# 560835; RRID: AB_2033956
Mouse anti human CD25 PE (Clone M-A251)	BD Biosciences	Cat# 555433; RRID: AB_395827
Mouse anti human CD25 PE-Cy5 (Clone M-A251)	BD Biosciences	Cat# 555433; RRID: AB_395827
Mouse anti human FoxP3 Alexa Fluor 647 (Clone 259D/C7)	BD Biosciences	Cat# 560045; RRID: AB_1645411
Mouse anti human FoxP3 PE (Clone 259D/C7)	BD Biosciences	Cat# 560046; RRID: AB_1645508
Mouse anti human FoxP3 PE-CF594(Clone 259D/C7)	BD Biosciences	Cat# 562421; RRID: AB_11153143
Mouse anti human FoxP3 Horizon V450(Clone 259D/C7)	BD Biosciences	Cat# 560459; RRID: AB_1645591
PE-Cy™7 Mouse Anti-Human CD69 (Clone FN50)	BD Biosciences	Cat# 560712; RRID: AB_1727509
Mouse Anti-Human CD45RA APC-H7 (Clone HI100)	BD Biosciences	Cat# 560674; RRID: AB_1727497
Mouse anti human TNFα APC (clone MAb11)	BD Biosciences	Cat# 551384; RRID: AB_2204110
Mouse anti human IFNγ PE-Cy7(clone B27)	BD Biosciences	Cat# 557643; RRID: AB_396760
Mouse anti human CD152 (CTLA4) APC (Clone BNI3)	BD Biosciences	Cat# 555855; RRID: AB_398615
Mouse anti-NF-κB p65 (pS529) Alexa Fluor 647	BD Biosciences	Cat# 558422; RRID: AB_647136
Mouse anti-Stat3 (pS727) Alexa Fluor 647	BD Biosciences	Cat# 558099; RRID: AB_397024
Mouse anti-Stat 5 (pY694) Alexa Fluor 647	BD Biosciences	Cat# 612599; RRID: AB_399882
Mouse anti-mTOR (pS2448) Alexa Fluor 647	BD Biosciences	Cat# 564242; RRID: AB_2738695
Mouse anti-CD247 (CD3ζ) (pY142) Alexa Fluor 647	BD Biosciences	Cat# 558489; RRID: AB_647152
Alexa Fluor 647 Mouse IgG1 κ Isotype control	BD Biosciences	Cat# 557783; RRID: AB_396871
PE/Cyanine7 anti-human CD39 (clone A1)	BioLegend	Cat# 328212; RRID: AB_2099950
PerCP/Cyanine5.5 anti-human CD73 (clone AD2)	BioLegend	Cat# 344014; RRID: AB_2561757
PE/Cyanine7 anti-human CD279 (PD-1) (clone EH12.2H7)	BioLegend	Cat# 329918; RRID: AB_2159324
Brilliant Violet 711(TM) anti-human CD223 (LAG-3) (Clone 11C3C65)	BioLegend	Cat# 369320; RRID: AB_2716125
Akt/PKB (pS473) Alexa Fluor 647	Cell Signaling Technology	Cat#4075; RRID: AB_916029
NF-κB p65 (pS536) Alexa Fluor 647	Cell Signaling Technology	Cat# 4887; RRID: AB_561198
S6-Ribos. Prot. (pS235/236) Alexa Fluor 647	Cell Signaling Technology	Cat# 4851; RRID: AB_10695457
Rabbit (DA1E) mAb IgG Isotype Control (Alexa Fluor 647 Conjugate)	Cell Signaling Technology	Cat# 2985; RRID: AB_1196589
Human Fc Receptor Binding Inhibitor Polyclonal Antibody	Thermo Fisher Scientific	Cat# 14-9161-73; RRID: AB_468582
Biotin Conjugated Anti-human CD28 (costimulatory) clone CD28.2	Thermo Fisher Scientific	Cat# 13-0289-82; RRID: AB_466415
Biotin Conjugated Anti-human CD2 (LFA-2) clone RPA-2.10	Thermo Fisher Scientific	Cat# 13-0029-82; RRID: AB_466314
Biotin Conjugated Anti-CD3 (Clone OKT3)	Taskén lab	N/A
FoxP3 antibody rabbit polyclonal	Novus Biologicals	Cat# NB600-245; RRID: AB_10001076
APC-H7 Rat anti-Mouse CD4 (Clone GK1.5)	BD Biosciences	Cat# 560181; RRID: AB_1645235
BV786 Rat Anti-Mouse CD8a (Clone 53-6.7)	BD Biosciences	Cat# 563332; RRID: AB_2721167
PE Rat Anti-Mouse Foxp3	BD Biosciences	Cat# 560408; RRID: AB_1645251
Fixable Viability Stain 780	BD Biosciences	Cat# 565388; RRID: AB_2869673
Fixable Viability Stain 700	BD Biosciences	Cat# 564997; RRID: AB_2869637
PerCP/Cyanine5.5 anti-mouse CD3 (Clone 17A2)	BioLegend	Cat# 100218; RRID: AB_1595492
APC anti-mouse CD45 (clone 30-F11)	BioLegend	Cat# 103112; RRID: AB_312977
PE/Cyanine5 anti-mouse CD69 (clone H1.2F3)	BioLegend	Cat# 104510; RRID: AB_313113
InVivoPlus rat IgG2a isotype control	Bio X Cell	Cat# BP0089; RRID: AB_1107769
InVivoPlus anti-mouse PD-1 (CD279)	Bio X Cell	Cat# BP0146; RRID: AB_10949053

**Bacterial and virus strains**		

*Escherichia coli* Rosetta (DE3)	Taskén lab	N/A

**Biological samples**		

Human buffycoats	Oslo University Hospital Blood Bank	Ethics committee approval #28075
Chronic lymphocytic leukemia (CLL) patient PBMCs	Oslo University Hospital	Ethics committee approval #2016/947
Lymph nodes (from patients with breast cancer with metastasis to regional lymph nodes)	Oslo University Hospital	Ethics committee approval #254844

**Chemicals, peptides, and recombinant proteins**		

Primaquine bisphosphate	Sigma-Aldrich	Cat# 160393-1G
Hydroxychloroquine sulfate	Sigma-Aldrich	Cat# H0915-5MG
Chloroquine diphosphate salt	Sigma-Aldrich	Cat# C6628-25G
Amodiaquin dihydrochloride dihydrate	Sigma-Aldrich	Cat# A2799-5G
Dipyridamole	Sigma-Aldrich	Cat# D9766-1G
Carbamazepine	Sigma-Aldrich	Cat# C4024-1G
Triamterene	Sigma-Aldrich	Cat# T4143-10G
Quinacrine dihydrochloride dihydrate	Santa Cruz Biotechnology	Cat# sc-391946
Amiloride HCl dihydrate	Selleckchem	Cat# S2560-50mg
Staphylococcal enterotoxin B	Sigma-Aldrich	Cat# S4881-1MG
Compounds from MolPort	MolPort	Table S1
His-FoxP3	This manuscript	N/A
His-FoxP3-ΔN (182-421aa)	This manuscript	N/A
Recombinant Human IL-2 Protein	R&D systems	Cat# 202-IL-050
DyLight™ 594 NHS Ester	Thermo Fisher Scientific	Cat# 46412
Alexa Fluor™ 750 NHS Ester (Succinimidyl Ester)	Thermo Fisher Scientific	Cat# A37575
Brefeldin A	Sigma-Aldrich	Cat# B5936
Phorbol 12-myristate 13-acetate (PMA)	Sigma-Aldrich	Cat# P8139
Ionomycin	Sigma-Aldrich	Cat# I0634
Kolliphor HS15	Sigma-Aldrich	Cat# 42966
Avidin	Thermo Fisher Scientific	Cat# 434401
SYBR Safe DNA Gel Stain	Thermo Fisher Scientific	Cat# S33102

**Critical commercial assays**		

Lymphoprep	STEM CELL technologies	Cat# 07861
RosetteSep™ T cell enrichment kits	STEM CELL technologies	Cat# 15061
Human CD4^+^CD25^+^CD127dim/- Regulatory T cell Isolation Kit II	Miltenyi Biotec	Cat# 130-094-775
T cell Activation/Expansion Kit, human	Miltenyi Biotec	Cat# 130-091-441
AlphaScreen Histidine (Nickel Chelate) Detection Kit	PerkinElmer	Cat# 6760619C
CM5 senor chip	Cytiva	Cat# BR100530
Direct-zol RNA Miniprep Kits	Zymo Research	Cat# R2050
cDNA using First Strand cDNA synthesis kit	Thermo Fisher Scientific	Cat# K1612
CellTrace™ Far Red Cell Proliferation Kit	Thermo Fisher Scientific	Cat# C34564
Phosflow Perm Buffer III	BD Biosciencess	Cat# 558050
Human FoxP3 buffer set	BD Biosciencess	Cat# 560098; RRID: AB_2869302
Mouse Foxp3 Buffer Set	BD Biosciencess	Cat# 560409; RRID: AB_2869340

**Experimental models: Cell lines**		

Panc02	ATCC	RRID: CVCL_D627

**Experimental models: Organisms/strains**		

C57BL/6NTc mouse	Taconic	Black 6 (B6NTac)
Hsd: Athymic Nude-Foxn1^nu^ mouse	In house breeding	RRID: IMSR ENV: HSD-069

**Oligonucleotides**		

Primers for ChIP qPCR FoxP3-Fwd: 5′-TTGCCCAGATTTTTCCGCC-3′	This manuscript	N/A
Primers for ChIP qPCR FoxP3-Rev: 5′-TCTCGGAACGAAACCTGTG-3′	This manuscript	N/A
Primers for ChIP qPCR STAT3-Fwd: 5′-AGCCAAGAGGAGACTGATAC-3′	This manuscript	N/A
Primers for ChIP qPCR STAT3-Rev: 5′-GCATTTAAAGTGCCTTGACG-3′	This manuscript	N/A
Primers for ChIP qPCR CD25-Fwd: 5′-AAATCAGGCTGTAAACAGAGG-3′	This manuscript	N/A
Primers for ChIP qPCR CD25-Rev: 5′-GGAGTTTTGGGGATGACAC-3′	This manuscript	N/A
DRE3 5′-AGATAAACAAGTGTAAACAAT-3′	Li et al.^45^	N/A
DNA for AlphaScreen 5′-Biotin-TGACAGTCAGCAAGGTAAAC AAGAGTAAACAAGTCCTGAGTCAGT-3′	Koh et al.^46^	N/A
Primers for qPCR	This manuscript	Table S2

**Recombinant DNA**		

PET-30a-FoxP3	This manuscript	N/A
PET-30a-FoxP3-ΔN (182-421aa)	This manuscript	N/A

**Software and algorithms**		

FlowJo	FlowJo	https://www.flowjo.com/
Cytobank	Beckman Coulter	https://cellmass.cytobank.org
GraphPad Prism 10	GraphPad	https://www.graphpad.com/

### Experimental model and study participant details

#### Primary human T cells

Buffy coats from human healthy donors were ordered from Oslo University Hospital Blood Bank (Oslo, Norway) with approval from the Regional Ethics Committee (REK #28075) and donor consent. CD3^+^ T cells were isolated from buffy coats using RosetteSep T cell enrichment kits (STEM CELL technologies) followed by gradient separation using Lymphoprep (STEM CELL technologies) according to the manufacturer’s manual. Purification of Treg cells was performed by magnetic separation using Human CD4^+^CD25^+^CD127^dim/-^ Regulatory T cell Isolation Kit II (Miltenyi Biotec) according to the instructions from the manufacturer. During the purification, Treg-depleted CD4 Teff and CD8 Teff cells were collected for the following assays. Cells were cultured in complete medium: RPMI 1640 medium supplemented with 10% fetal calf serum (FCS), nonessential amino acid (NEAA), Sodium Pyruvate and 1% penicillin-streptomycin (Thermo Fisher Scienific) at 37°C incubator with 5% CO2.

#### Patient samples

Representative LN biopsies from patients with breast cancer with metastasis to regional lymph nodes were collected at surgery and dissociated into viable cell suspensions as described.^37^ Isolation and preservation of PBMCs from CLL patients were as described previously.^47^ Cryopreservation of CLL cells has been shown not to affect their functionality.^48^ Frozen cells were thawed in complete RPMI medium supplemented with 50% FBS and washed once with complete medium. Cells were re-suspended in IMDM+ 5% human serum (Merck KGaA, Darmstadt, Germany) and 10U IL-2 to recover for 30 min before compound treatment. For each patient sample, 2×10^5^ cells of breast cancer patient LN samples and 3×10^6^ PBMCs from CLL samples per well were distributed into 96-well plates and treated with compounds for 48 h. 10 μg/mL BFA was added to the culture 6 h before harvesting. Cells were stained with Fixable Viability Stain 700, followed by a block with human Fc Receptor Binding Inhibitor Polyclonal Antibody (Invitrogen) and subjected to a flow cytometry antibody staining protocol as above.

The studies of patient samples were approved by the Regional Committee for Medical and Health Research Ethics for South-East Norway (REK approval #254844 for breast cancer AXL study and #2016/947 for CLL study). Prior to sample collection, all patients signed a written informed consent. Research on blood samples was carried out in agreement with Declaration of Helsinki.

#### Mice experiments

Wild-type female C57BL/6 or nude (Hsd: Athymic Nude-Foxn1nu) mice at 7–8 weeks of age were used in the experiments. For compound titration experiments, mice were administered with compounds by intraperitoneal injections every other day for up to 3 weeks, and terminated to harvest blood, and spleen at specified time points. For tumor growth experiments, 6 × 10^5^ or 1 × 10^6^ Panc02 cells were injected intradermally into the right flank of the WT mice or nude mice, respectively. Tumor sizes were measured every other day with a caliper. When the tumors were visible and measurable, mice were treated with compounds every other day by intraperitoneal injections for a total of 7 doses administered. Tumor volume was calculated using the formula: 0.5 × length × width^2^. All the compounds used for injections were formulated in 20% Kolliphor HS15 (Merck KGaA, Darmstadt, Germany) in Milli-Q water followed by sonication at 65°C for 15 min as described [34], with 20% Kolliphor HS15 in Milli-Q water as Vehicle control. InVivoPlus rat IgG2a isotype control and InVivoPlus anti-mouse PD-1 (CD279) were purchased from BioXcell. For the harvesting experiments, tumors, spleens and blood were collected and processed to get cells in suspension before staining for flow cytometry analysis. All mice experiments were conducted at the Department of Comparative Medicine, Oslo University Hospital, with approval from the Norwegian Food Safety Authority.

### Method details

#### High-throughput drug screen

CD3^+^ T cells isolated from healthy donors were distributed into 384-well V-bottom plate (3×10^5^ cells per well) by CERTUS FLEX dispenser (Fritz Gyger, Switzerland) and treated with compounds at 10μM for 16h, including wells with Staphylococcal enterotoxin B (1 μg/mL) and DMSO controls. After treatment, cells were fixed and permeablized in human FoxP3 buffer (BD Biosciences) before antibody staining. CD4 PE-Cy7 and FoxP3 Horizon V450 antibodies were transferred into the plate by LABCYTE Echo 550 acoustic liquid handler (Beckman coulter). All the liquid handling procedures were performed by the Beckman i7 Automated Liquid Handler platform (Beckman Coulter). Stained cells were washed twice with PBS and analyzed on a BD LSR Fortessa cytometer (BD Biosciences). Flow cytometry data were analyzed in Cytobank (https://cellmass.cytobank.org). Candidates that affect FoxP3 expression level represented by change of FoxP3% in CD4^+^ T cells compared to DMSO were selected for further validation.

Compounds from the Prestwick library were obtained from the High-Throughput Chemical Biology Screening Platform, Center for Molecular Medicine Norway, University of Oslo. Amiloride HCl dihydrate was ordered from Selleckchem. Triamterene, dipyridamole, carbamazepine, primaquine bisphosphate, hydroxychloroquine sulfate, chloroquine diphosphate salt and amodiaquin dihydrochloride dihydrate were purchased from Sigma-Aldrich (Merck KGaA, Darmstadt, Germany). Quinacrine dihydrochloride dehydrate was ordered from Santa Cruz Biotechnology (Dallas, US). Compounds MP2 to MP25 were ordered from MolPort (Riga, Latvia), as listed in Table S1. Synthesis of MP4 was done at O2H (Ahmedabad, India).

#### *In-silico* analysis

Based on the initial screening, several rounds of analogs of hit compounds were identified in the MolPort (https://www.molport.com/) and ZINC (http://zinc15.docking.org/) databases. These compounds were selected based on similarity and/or sub-5 structure searches using the Radial (ECFP) binary fingerprint and a Tanimoto coefficient cut-off of >0.5. A Similarity/Distance screen was performed to create the compounds' Similarity/Distance matrix. For structure-activity relationship (SAR), R-group analysis was performed. The pan-assay interference compounds (PAINS) filter was applied to remove chemical compounds often giving false positive results in high-throughput screens. A selection of such analogs was ordered from MolPort. All the computational chemistry tasks were performed using Canvas (version 4.2.012), part of the Schrodinger Suite (release 2019.4), using the SDF files downloaded from MolPort.

#### T cell proliferation and Treg suppression assay

For cell proliferation assays, 2 × 10^5^ cells were stained with CellTrace Far Red (Invitrogen, Thermo Fisher Scientific) at 2 μM for 20 min at 37°C. Complete medium was then added to quench the staining for 5 min. After washing with complete medium, the cells were cultured in complete medium and stimulated with human CD2/CD3/CD28 beads (Miltenyi Biotec, T cell activation/expansion kit) at a 1:5 ratio (beads:cells) for 96 h with or without compounds treatment. Harvested cells were then washed with PBS and stained with Fixable Viability Stain 780 (BD Biosciences, Franklin Lakes, US) before Flow cytometry analysis on BD LSR Fortessa cytometer (BD Biosciences, Franklin Lakes, US) to measure the percentage of CellTrace Far Red-positive cells that represent the ratio of cell proliferation.

For Treg suppression assays, 1 × 10^5^ isolated Tregs were pre-activated with human CD2/CD3/CD28 beads for 48 h in the absence or presence of compounds. In the simplified assay for compound screening, Tregs were removed of compounds and re-suspended in fresh medium to be co-cultured with 2 × 10^5^ CellTrace Far Red-stained Teffs. In the protocol for detailed investigation, Tregs treated with compounds were washed once with fresh medium and re-counted to ensure a 1:2 ratio with Teffs in co-culture. The co-cultured cells were then stimulated for 96 h with human CD2/CD3/CD28 beads at a 1:5 ratio of beads:cells. Proliferating CellTrace Far Red-positive cells were then measured by flow cytometry. The level of suppression of Teffs by Tregs was determined by the difference in the proliferation of Teffs in co-culture with Tregs compared to Teffs alone.

#### Flow cytometry analysis

T cells were first washed with PBS and stained with Fixable Viability Stain, followed by fixation and permeabilization with FoxP3 buffer set (BD Bioscience, Franklin lakes, US) before antibody staining for flow cytometry analysis. For the primary screening, T cells were stained with CD4 PE-Cy7 and FoxP3 Horizon V450 antibodies for flow staining. For Treg surface marker staining, cells were after treatment stained with Fixable Viability Stain 700 (BD Bioscience), followed by staining of surface proteins and fixation in FoxP3 buffer before flow cytometry analysis. For intracellular cytokine measurements, Phorbol 12-myristate 13-acetate (PMA, 50 ng/mL), Brefeldin A (BFA, 10 μg/mL), and Ionomycin (1 μg/mL) (Merck KGaA, Darmstadt, Germany) were added to the cell culture for 4 h at 37°C before harvesting for viability and antibody staining for flow cytometry. All flow cytometry experiments were performed on a BD LSR Fortessa cytometer (BD Biosciences, Franklin lakes, US) configured with 488nm, 561nm, 640nm and 407nm lasers. The flow data were analyzed by FlowJo v10.

#### Phospho-flow signaling analysis

CD3^+^ T cells were treated with the indicated compounds for 30 min at 37°C and analyzed for multiple phospho-epitopes by our phospho-flow protocol as described.^18^^,^^49^ For TCR activation analyses, cell suspensions in RPMI with 1% FCS were treated with QDD or MP4 for 30 min, followed by stimulation with biotinylated anti-CD3 (1 μg/mL, OKT3), anti-CD2 (5 μg/mL, RPA-2.10), anti-CD28 (5 μg/mL, CD28.2) and Avidin (20 μg/mL) (eBioscience) for the indicated time points at 37°C and subjected to immediate fixation with BD Phosflow Fix Buffer I (BD Bioscience) for 10 min at 37°C. After washing with PBS, fixed cells were barcoded in serial dilutions of combinations of DyLight 594 NHS Ester (Thermo Fisher Scientific) and Alexa Fluor 750 NHS Ester (Succinimidyl Ester) (Thermo Fisher Scientific) for 20 min at room temperature. After washing to remove excess dye, barcoded cells were pooled together and permeabilized in FoxP3 buffer C for 30 min at room temperature, followed by a secondary permeabilization in BD Phosflow Perm Buffer III (BD Bioscience) for at least 30 min at −80°C. At last, the samples were washed and stained with antibodies against CD4, CD8, CD25, FoxP3, and Ax647-conjugated phosphor-antibodies before analysis on a BD LSR Fortessa instrument. For IL-2 stimulation, CD3^+^ T cells were treated with compounds for 30 min before stimulation with 50 U IL-2 (R&D systems) at the indicated time points, followed by the same staining protocol as above. Phospho-Flow cytometry data were analyzed in Cytobank (https://cellmass.cytobank.org). Protein phosphorylation change was presented as arcsinh ratio of Ax647 MFI,^50^ using algorithm in Cytobank.

#### Protein purification

Human full-length FoxP3 or FoxP3-ΔN (182-421aa) were cloned into the pET-30a vector (Novagen) at restriction sites BamHI and XhoI. Transformed Rosetta cells were then induced with 0.1mM IPTG (Thermo Fisher Scientific) at 18°C for 16 h, followed by purification. In brief, collected cell pellets were sonicated in lysis buffer (50mM Na3PO4/Na2HPO4 pH7.8, 300 mM NaCl) with EDTA-free protease inhibitors (Complete Mini, Roche). After centrifugation, the supernatant was collected and incubated with HIS-select Nickel Affinity gel (Millipore) at 4°C overnight. Then, the protein-bound Nikkel gel was first washed with lysis buffer containing 20mMimidazole twice and then washed with lysis buffer containing 40mM imidazole, followed by three times elution with lysis buffer containing 500mM imidazole. Mixed protein eluates were next dialyzed overnight at 4°C using a 10KD Dialysis Cassette (Thermo Fisher Scientific) in dialysis buffer (20mM Tris-HCl pH 7.5, 150mM NaCl, 0.25mM DTT). The purity and concentration of His-FoxP3 proteins were then determined by SDS PAGE gel.

#### Electrophoretic mobility-shift assays (electrophoretic mobility shift assays)

The FoxP3 binding DNA sequence DRE3 (5′-AGATAAACAAGTGTAAACAAT-3′)^45^ was used in the EMSA assay. Single-strand DNA was denatured at 94°C for 5 min, and then allowed to anneal with its complementary strand while cooling down to room temperature before use. Binding assays were performed by incubating 3 μg purified His-FoxP3- ΔN protein and 20nM dsDNA for 30 min at room temperature within reaction buffer (10 mM Tris pH 7.5, 100 mM KCl, 1 mM EDTA, 0.1 mM DTT, 5% v/v glycerol, 0.010 mg/mL BSA) at a total volume of 20μL. For compound-competition assay, His-FoxP3-ΔN protein was pre-incubated with compounds for 30 min at room temperature before mixing with DNA. When the reaction was done, 6×DNA loading buffer was added, and the samples were run on 10% Criterion TGX gel (Bio-Rad) in TAE buffer (Bio-Rad Laboratories), 100V for 1h. Afterward, the gel was stained with 1× SYBR Safe DNA Gel Stain (Thermo Fisher Scientific) for 30 min at room temperature, followed by imaging with GelDoc system (Bio-Rad Laboratories).

#### Surface plasmon resonance analysis

All SPR analyses were performed by Biacore T200 (Cytiva) at 25°C. Purified His-FoxP3 was immobilized on a CM5 sensor chip (Cytiva) in flow cell 2, with flow cell 1 as the blank control. 1X HBS-EP+ buffer (0.01 M HEPES, 0.15 M NaCl, 0.003 M EDTA and 0.05% v/v Surfactant P20) was used for immobilization.

For assessing compound binding to FoxP3, the LMW screening method supplied in the Biacore software was applied according to the manufacturer’s instructions, using PBS+0.05% Tween 20 + 2% DMSO as the running buffer. In brief, 50 μM of each compound was injected at 30 μL/min with 60 s contact time and 300 s dissociation time. After each compound injection, the system was washed with 50% DMSO. Serial dilutions of DMSO at 2%–3% range were used for solvent correction to normalize variations in the bulk response. The affinity between compound and FoxP3 protein were analyzed afterward. For affinity kinetic measurements, a range of concentrations of QDD or MP4 was injected and allowed to bind to the FoxP3 protein on the chip and steady state affinity constants (Rmax) were assessed for comparison.

#### AlphaScreen assay on FoxP3-DNA interaction

AlphaScreen assays were performed to measure the effects of compounds on FoxP3-DNA binding. Purified His-FoxP3-ΔN protein and an annealed biotinylated-FoxP3 binding double-stranded DNA sequence (5′-Biotin-TGACAGTCAGCAAGGTAAACAAGAGTAAACAAGTCCTGAGTCAGT-3′)^46^ were used in the assay at a 2:1 ratio. The assay was performed following the manufacturer’s instruction provided for the AlphaScreen Histidine (Nickel Chelate) Detection Kit (PerkinElmer, Waltham, US), with the whole reaction volume at 20 μL in 384-alpha plates (PerkinElmer, Waltham, US). First, FoxP3 protein, DNA and compound at different concentrations were incubated in the reaction buffer at room temperature for 1 h. Then, acceptor beads were added to the reaction and incubated for 1 h, followed by the addition of donor beads for another 1 h-incubation. His-Biotin incubated with compounds at the same concentrations as in FoxP3-DNA reactions were included as counter screen. The luminescence signals were subsequently measured in an Envision plate reader (PerkinElmer, Waltham, US) with an AlphaScreen filter installed. The effect of compounds in inhibiting FoxP3-DNA binding was calculated by normalization of the signal in FoxP3-DNA reactions to that of the counter screen and displayed as IC50.

#### Quantitative PCR (qPCR)

CD3^+^ T cells were treated with compounds for 24 h before RNA isolation. Total RNA was extracted using Direct-zol RNA Miniprep Kits (Zymo Research, Irvine, US). 1μg total RNA was reverse transcript to cDNA using First Strand cDNA synthesis kit (Thermo Fisher Scientific), followed by real-time PCR assays performed in Applied Biosystem7500 Real-Time PCR system (Applied Biosystems, Waltham, US), using SYBR green master mix (Applied Biosystems) and indicated primers. Quantitative gene expression analysis was calculated using the 2^−ΔΔCT^ method with internal RPS9 control, and the fold-change of compound-treated samples was obtained by normalization to mock control. Primers for qPCR are listed in Table S2.

#### Chromatin-immunoprecipitation (chromatin immunoprecipitation) qPCR

ChIP experiments were performed by modifying the cross-linking protocols as described.^51^ In brief, a total of 4×10^6^ isolated CD4^+^CD25^+^CD127^dim−^ Tregs pooled from 3 healthy donors were stimulated with CD2/CD3/CD28 beads for 3 days and treated with indicated compounds for 4 h. Then the cells were cross-linked with 1% formaldehyde for 10 min which was quenched by adding 125 mM Glycine. After washing with PBS, cell pellets were collected for nuclei isolation. Cells were first re-suspended in Lysis Buffer I (50mM Hepes-KOH pH 7.5, 140mM NaCl, 1mM EDTA, 10% glycerol, 0.55% NP-40, 0.25% Triton X-100) for 1h rotation at 4°C, and collected for pellets after 2000 g centrifugation for 5min. Pellets were then re-suspended in Lysis Buffer II (200 mM NaCl, 1mM EDTA, 0.5Mm EGTA, 10mM Tris-HCl pH7.5) for 4 min rotation at 4°C, and the nuclei in pellet were collected after centrifugation at 2000*g* for 5 min. Isolated nuclei were re-suspended in Lysis Buffer III (1 mM EDTA, 0.5 mM EGTA, 10 mM Tris-HCl pH7.5, 100 mM NaCl, 0.1% Na-Deoxycholate, 0.5% N-lauroyl sarcosine) followed by sonication using Bioruptor Twins (Diagenode, Liege, Belgium) with 30 s ON and 30 s OFF for 35 cycles to get DNA fragments of around 200-bp size. After centrifugation, 1% of the supernatant volume was kept as an input control. The remaining part of each sample was first pre-cleared by incubating with rabbit IgG coupled protein G beads (Thermo Fisher Scientific, Waltham, US) for 1h at 4°C. After removing the beads, samples were then immunoprecipitated with anti-FoxP3 antibody (Novus Biologicals, Centennial, US) or Rabbit IgG control coupled protein G beads overnight at 4°C. After de-crosslinking, the antibody-enriched DNA was eluted and purified over a column (Macherey-Nagel, Düren, Germany) before running qPCR with primers targeting promoter regions of CD25, STAT3 and FoxP3 genes. The binding efficiency of FoxP3 antibody to DNA compared to IgG control was defined as % of input control. ChIP qPCR primers were designed using ChIP-seq data published.^52^

### Quantification and statistical analysis

One-way ANOVA, two-way ANOVA and T-tests were performed to determine *p*-values using Graph Prism (GraphPad Software Inc.). Observed differences were regarded significant when p˂0.05, and represented as ∗p˂0.05, ∗∗p˂0.01, ∗∗∗, p˂0.001, ∗∗∗∗p˂0.00001. ‘ns’ means no significant. Details of sample sizes and statistics are described in the figure legends.
