# Supplementary material for: Identification of a group of 9-amino-acridines that selectively downregulate regulatory T cell functions through FoxP3
Source: iScience. 2025 Jan 31;28(3):111931. doi: 10.1016/j.isci.2025.111931 (PMC11872463; doi:10.1016/j.isci.2025.111931)
Supplement: Document S1. Figures S1–S8, Tables S1, and S2 [file mmc1.pdf]

iScience, Volume *28*

Supplemental information

Identification of a group of 9-amino-acridines that selectively downregulate regulatory T cell functions through FoxP3

Qian Wei, Håvard Foyn, Johannes Landskron, Shixiong Wang, Inga Hansine Rye, Sigrid S. Skånland, Hege Elisabeth Giercksky Russnes, Jo Klaveness, Rafi Ahmad, and Kjetil Taskén

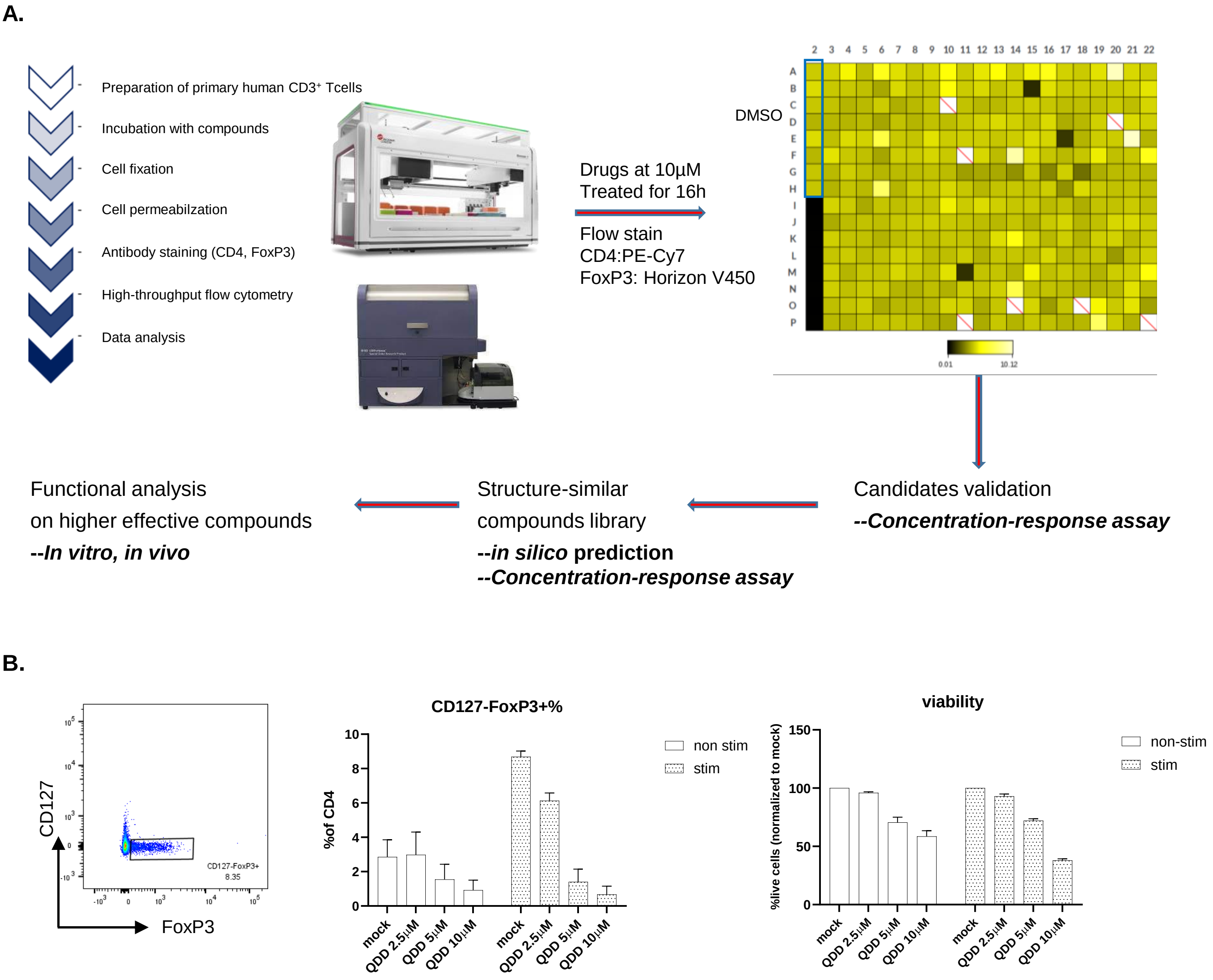

Figure S1. High-throughput drug screen on FoxP3 in T cells, related to Figure 1.

A) Workflow on the identification of novel small molecule regulators of FoxP3**.** Primary T cells isolated from healthy donors were treated with drugs at 10 µM for 16 h in 384-well V-bottom plates, followed by CD4 PE-Cy7 and FoxP3 Horizon V450 antibody staining and high-throughput flow cytometry analysis. Percentages of FoxP3 in CD4^{+} T cells were analyzed in Cytobank and are represented as a heatmap. Candidates potentially down-regulating FoxP3 were validated by concentration-response assays in T cells from 3 donors. A sub-library containing structurally similar compounds were assembled by in silico searches and validations of available compounds. Furthermore, the compounds that most effectively down-regulated FoxP3 were evaluated by functional analysis both in vitro and in vivo. **B)** CD3^{+} T cells from healthy donors were treated with compounds for two days with or without TCR stimulation to measure FoxP3 expression by flow cytometry analysis. CD127 versus FoxP3 were used in the gating together with viability stain to show the percentage of live cells. FoxP3 expression is represented by percentage of CD127^{-}FoxP3^{+} in CD4^{+} T cells. (n=3 donors) Graphs show Mean ±SEM.

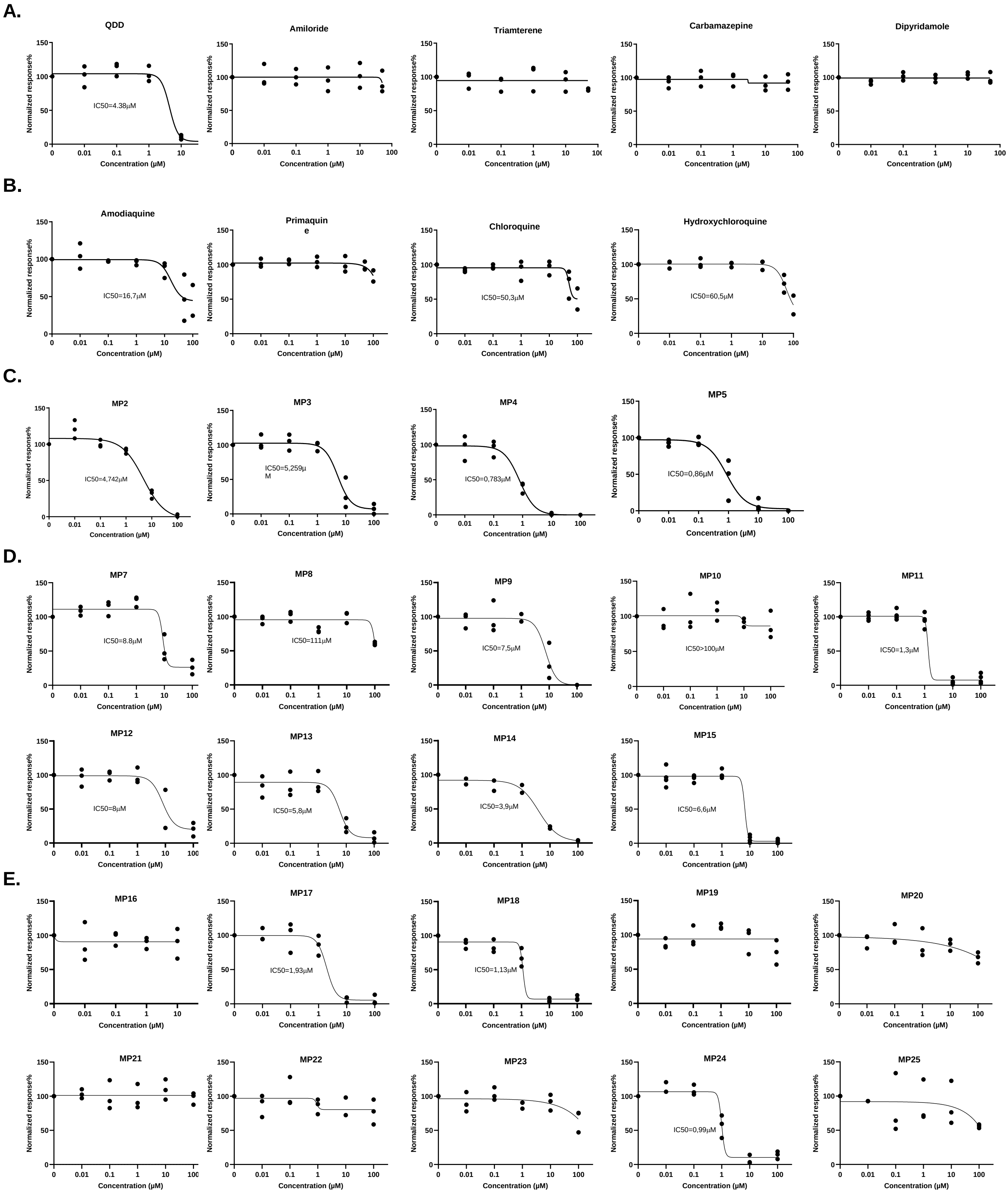

Figure S2. Dose-response curves of all compounds tested, related to Figure 1.

CD3^{+} T cells from healthy donors were treated with each compound at concentrations of 0-0.01-0.1-1-10-100 µM for 24 h, followed by flow cytometry analysis to measure FoxP3 expression in CD4^{+} T cells. Graphics represent results of compounds from group 0 **(A)**, group 1**(B)**, group 2 **(C)**, group 3 **(D)** and group 4 **(E)**. The response curve for each compound showed its effect on FoxP3 down-regulation by calculating IC50. Normalized response%: FoxP3% in CD4^{+} T cells in drug treated group normalized to that of mock treatment. (n=3 donors)

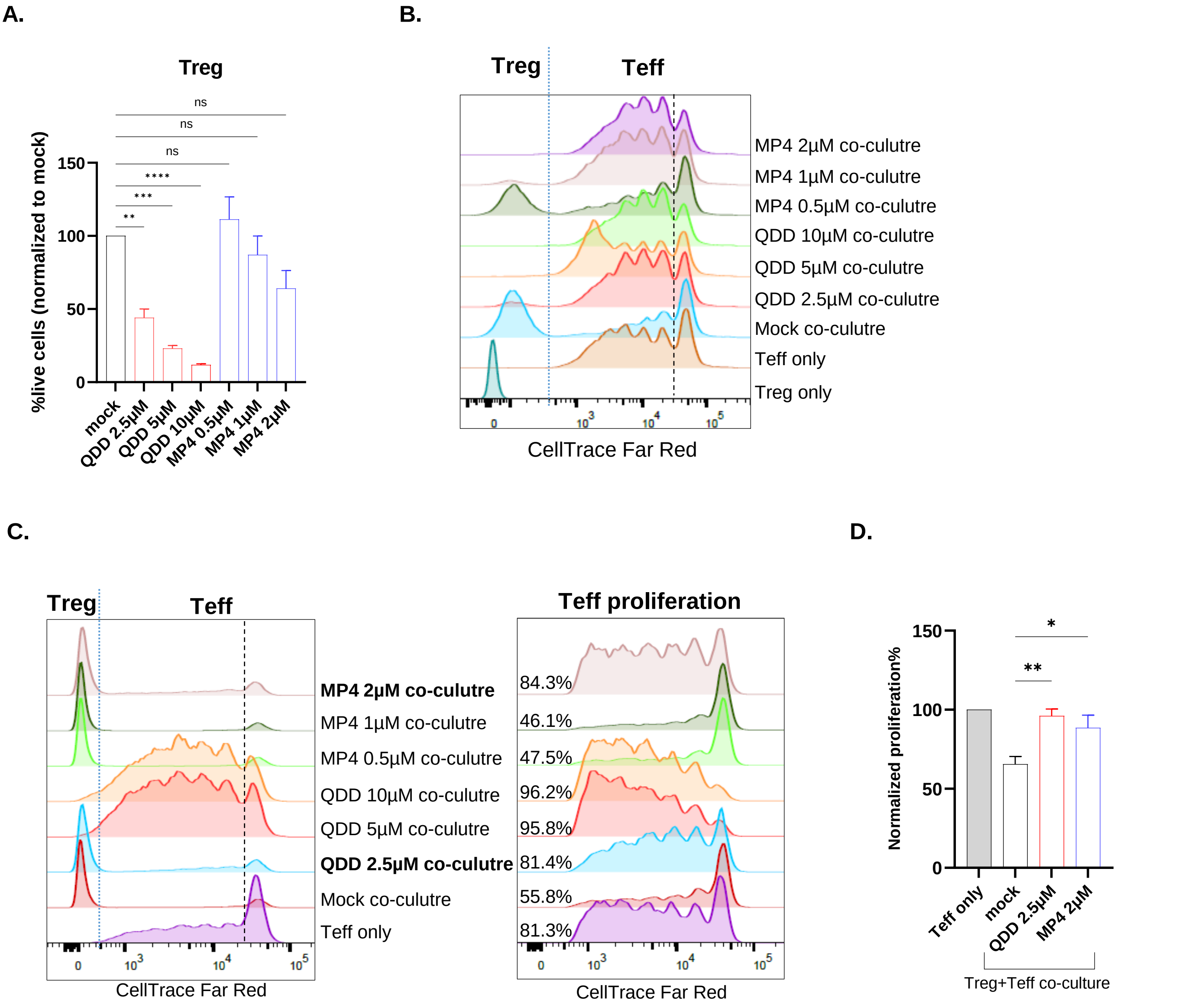

Figure S3. Effects of compounds on Treg suppressive functions, related to Figure 2.

Isolated Tregs were stimulated with TCR for two days together with compounds. **A)** Viability of Tregs after 2 days of compound treatment. **B)** Medium with compounds was removed from Tregs and was replaced by new medium without washing before co-culture with Teff. **C and D)** Tregs treated with compounds were washed once with medium and re-counted to have a Treg:Teff =1:2 ratio in the co-culutres. Dotted lines in the FACS histograms (**B an C**) represent the gatings to seperate Treg from Teff, and the Teff proliferation. Normalized proliferation (%) is calculated by setting the proliferation in Teff only as 100%. (n=3 donors). Graphs show Mean ±SEM (One-way ANOVA).

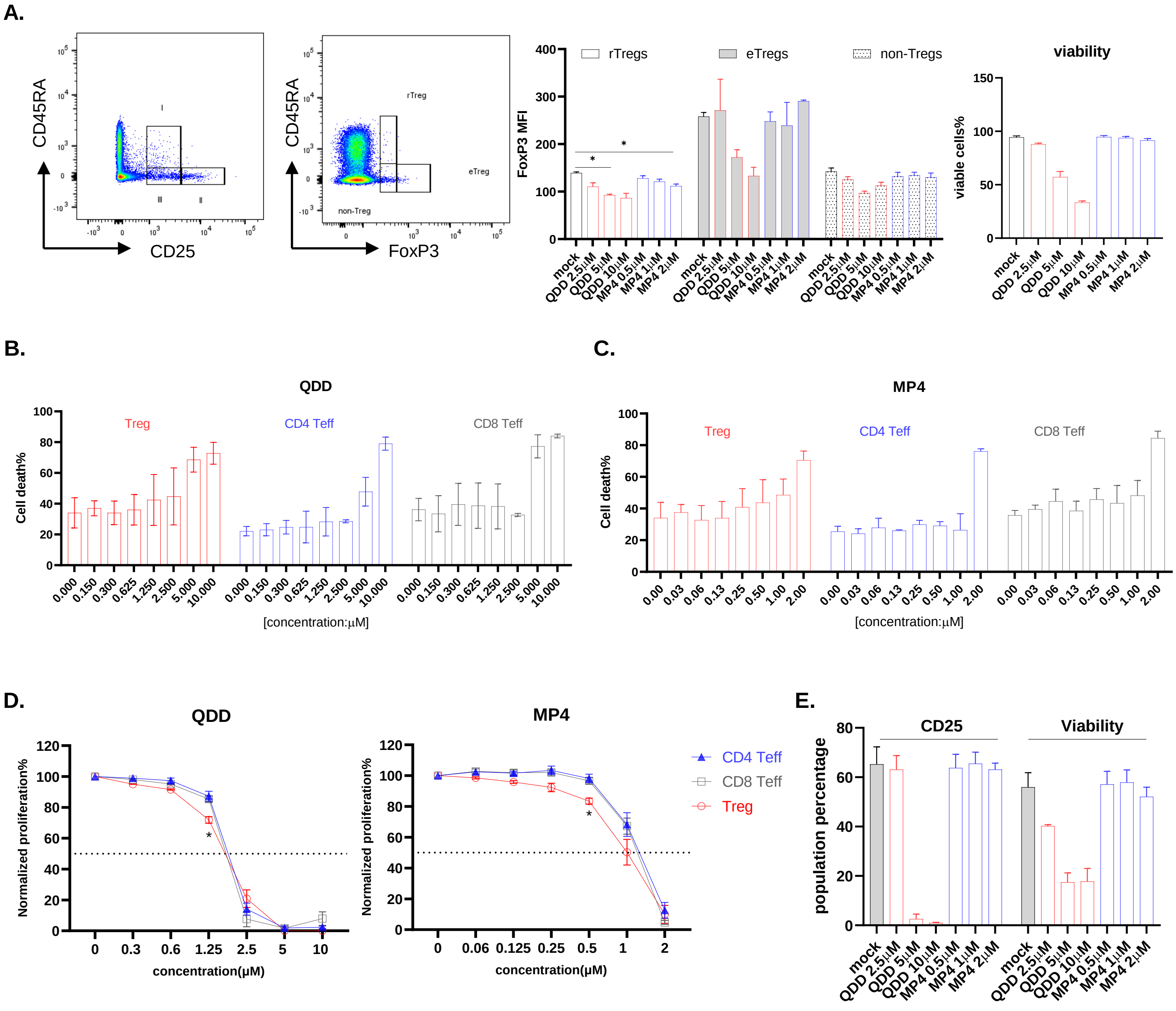

Figure S4. Toxicity and specificity of QDD and MP4 in T cells, related to Figure 3.

A) CD3$^+$ T cells from healthy donors were treated with compounds for 2 days without TCR stimulation. Different Treg populations were gated either using CD45RA versus CD25 (left FACS plot), or CD45RA vs FoxP3 (right FACS plot). FoxP3 expression in each Treg population is shown as MFI. **B and C)** Isolated CD4+Teff, CD8+ Teff and Tregs were stimulated with CD2/CD3/CD28 beads and treated with QDD **(B)** or MP4 **(C)** for four days. Cell death was determined by flow cytometry with staining of Fixable Viability Stain 700. **D)** CD3$^+$T cells from healthy donors were stained with CellTrace Far Red, followed by treatment with QDD or MP4 at indicated concentrations for four days under TCR stimulation. Cell proliferation of each population was determined by measuring the percentage of CellTrace Far Red-positive cells in flow cytometry analysis, together with gating using CD4, CD8, and FoxP3 antibodies. *represents the concentration where there is a significant difference between Treg and CD4/CD8 Teff. **(E)** Isolated CD4 Teffs were treated with indicated compounds for 48h under TCR stimulation. CD25% of CD4 and cell viability were analyzed by flow cytometry. (n=3 donors) Graphs show mean ±SEM (2-way ANOVA).

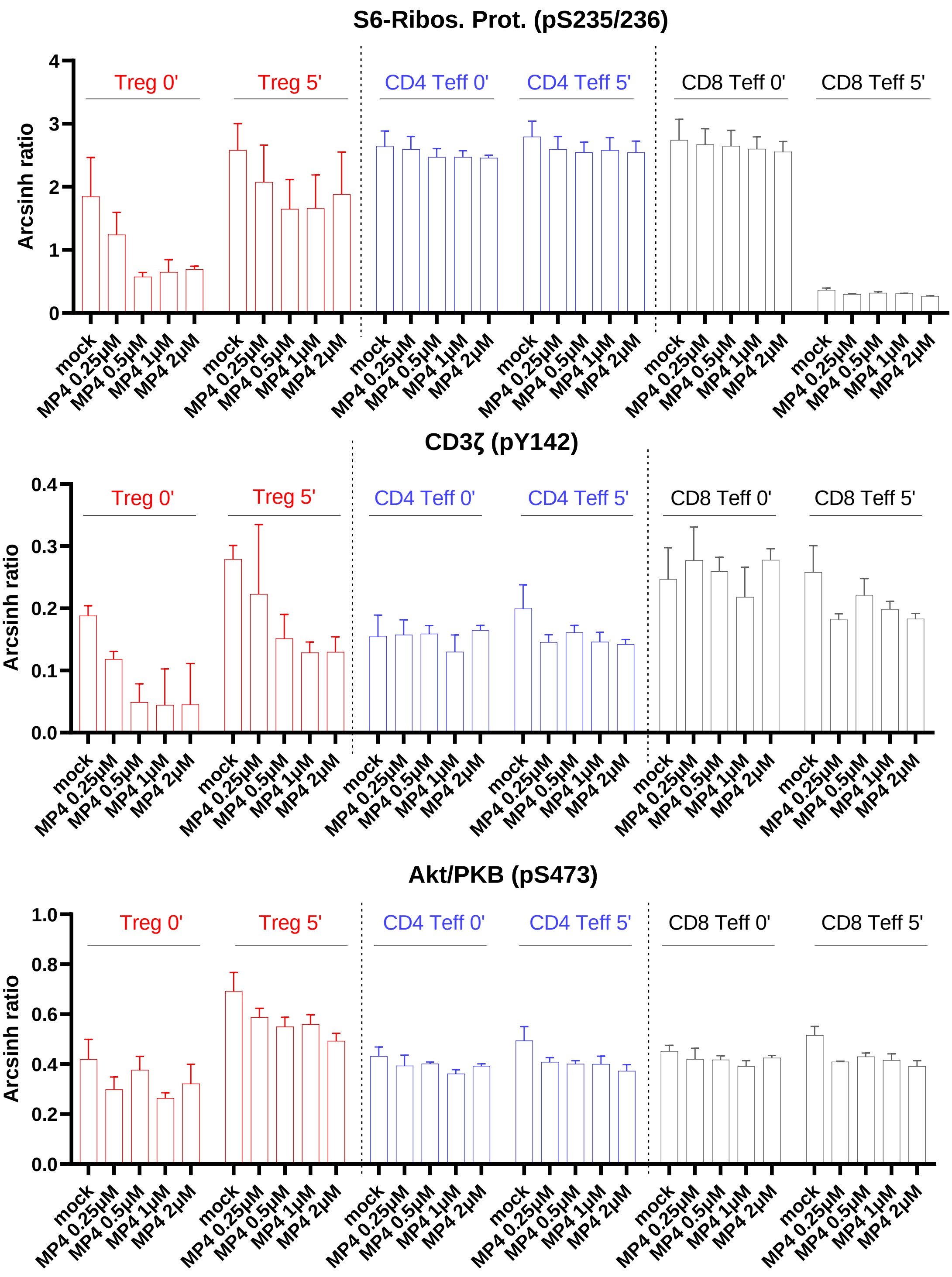

Figure S5. Representative protein phosphorylation in TCR signaling pathways in MP4-treated T cells, related to Figure 3.

CD3+ T cells isolated from healthy donors were pre-treated with MP4 at defined concentrations for 30 min without (0') or with subsequent stimulation by soluble anti-CD2/CD3/CD28 antibodies for 5 min (5') at 37°C. Phospho-flow was then performed to measure the phosphorylation levels of S6-ribosomal protein (pS235/236), CD3ζ (pY142) and AKT/PKB (pS473) in CD8 Teff ($CD8^+$), CD4 Teff ($CD4^+CD25^-FoxP3^-$) and Tregs ($CD4^+CD25^+FoxP3^+$) gated populations. Y-axis: Arcsinh ratio of MFI of each phosphorylation signal normalized to isotype control. Graphs show Mean ±SEM (n=3 donors).

A.

Compound	MW	IC50 on FoxP3 (µM)	RU value binding_early	RU value binding_late
Amiloride hydrochloride dihydrate	302,12	N/A	8,8	8,4
Triamterene	253,27	N/A	5,7	3,6
Carbamazepine	236,28	N/A	4,1	3,4
Dipyridamole	504,64	N/A	8	6,7
**QDD**	**508,91**	**4,38**	**204,2**	**227,4**
Amodiaquine	464,81	34,3	35,2	36,6
Primaquine	455,34	>100	6,3	6
Chloroquine	515,86	50,3	16,5	15,1
Hydroxychloroquine	433,95	60,5	27,8	25,3
MP2	230,7	4,742	37,8	40,3
MP3	340,85	5,259	11,4	13,1
**MP4**	**523,92**	**0,783**	**180,6**	**212,3**
MP5	262,356	0,86	26	27,1
MP7	393,46	8,8	109,3	114
MP8	285,299	111	19,4	35,4
MP9	343,383	7,5	536,1	585,4
MP10	285,299	>100	4,7	4,6
MP11	258,7	1,3	71	123
MP12	349,82	8	63,5	69,3
MP13	313,404	5,8	63,2	70,2
MP14	284,362	3,9	193,4	199,7
MP15	518,06	6,6	161,4	186
MP16	255,32	No response	33,1	38,8
MP17	252,317	1,93	51,4	74,6
MP18	276,383	1,13	88,5	96,8
MP19	266,3	No response	6,7	11
MP20	324,336	Bad response	4,5	7,8
MP21	280,327	No response	10,5	14,8
MP22	298,389	No response	7,8	18,5
MP23	270,335	Bad response	5,9	16,1
**MP24**	**441,4**	**0,99**	**177,8**	**207,7**
MP25	324,336	Bad response	3,5	4

B.

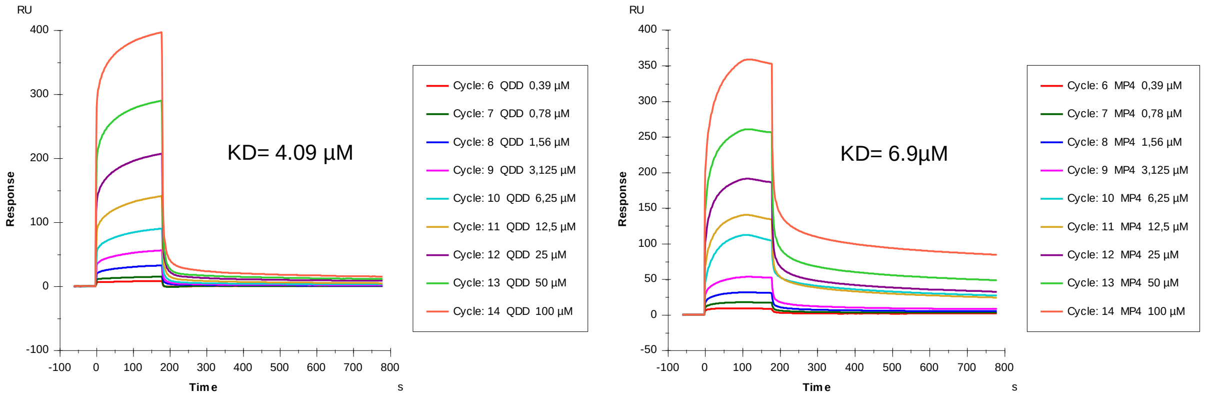

Figure S6. Affinity between FoxP3 and 9-amino-acridines by SPR analysis, related to Figure 4.

(A) LMW screen was performed on a Biacore T200 with His-FoxP3 on a CM5 chip and a group of 9-amino-acridines at 50 µM individually injected in the sample flow. The response unit (RU) value for binding early and late are listed in the table after subtraction of negative controls. **(B)** Direct binding between FoxP3 protein and QDD (left) or MP4 (right) was determined by SPR analysis, with His-FoxP3 immobilized on the CM5 chip. Affinity kinetics (KD) were calculated by 1:1 ratio affinity based on Rmax.

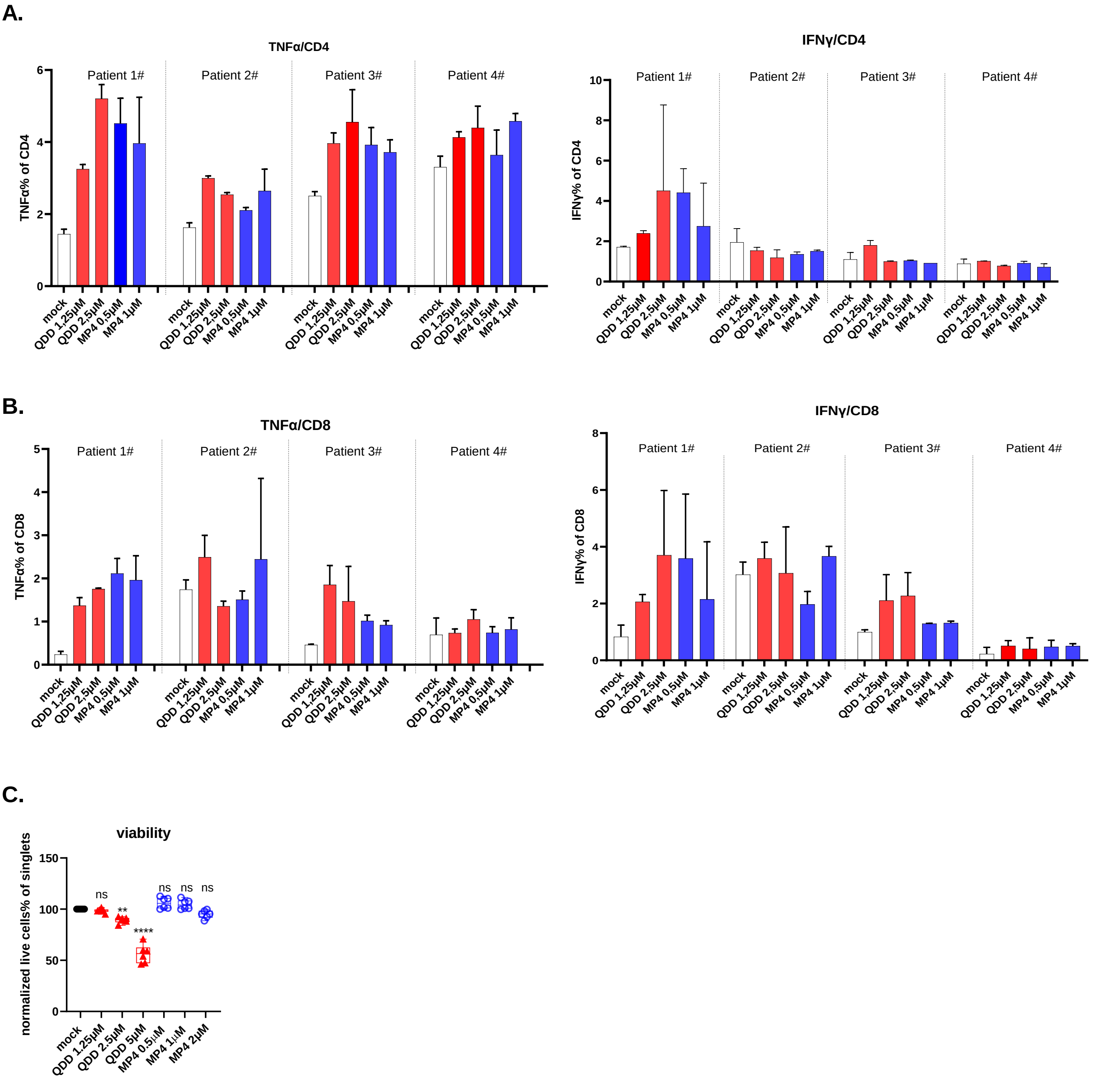

Figure S7. Effects of QDD and MP4 on boosting T cell activation in breast cancer patient LN samples, related to Figure 5.

Cell suspensions from breast cancer patient LN were treated with QDD or MP4 at specified concentrations for 48 h. BFA was added to the cultures 6 h before harvesting. Production of TNFα and IFNγ in CD4 **(A)** or CD8 **(B)** T cells were determined as the percentage of each population by intracellular cytokine staining analysed by flow cytometry. The plots show results from 4 individual patients, representing as mean± SD from 2 replicates. **(C)** Viability of the cells were determined by flow cytometry staining of Fixable Viability Stain 700. Graph shows all data points with median and Min to Max (n=6 patients, 2-way ANOVA).

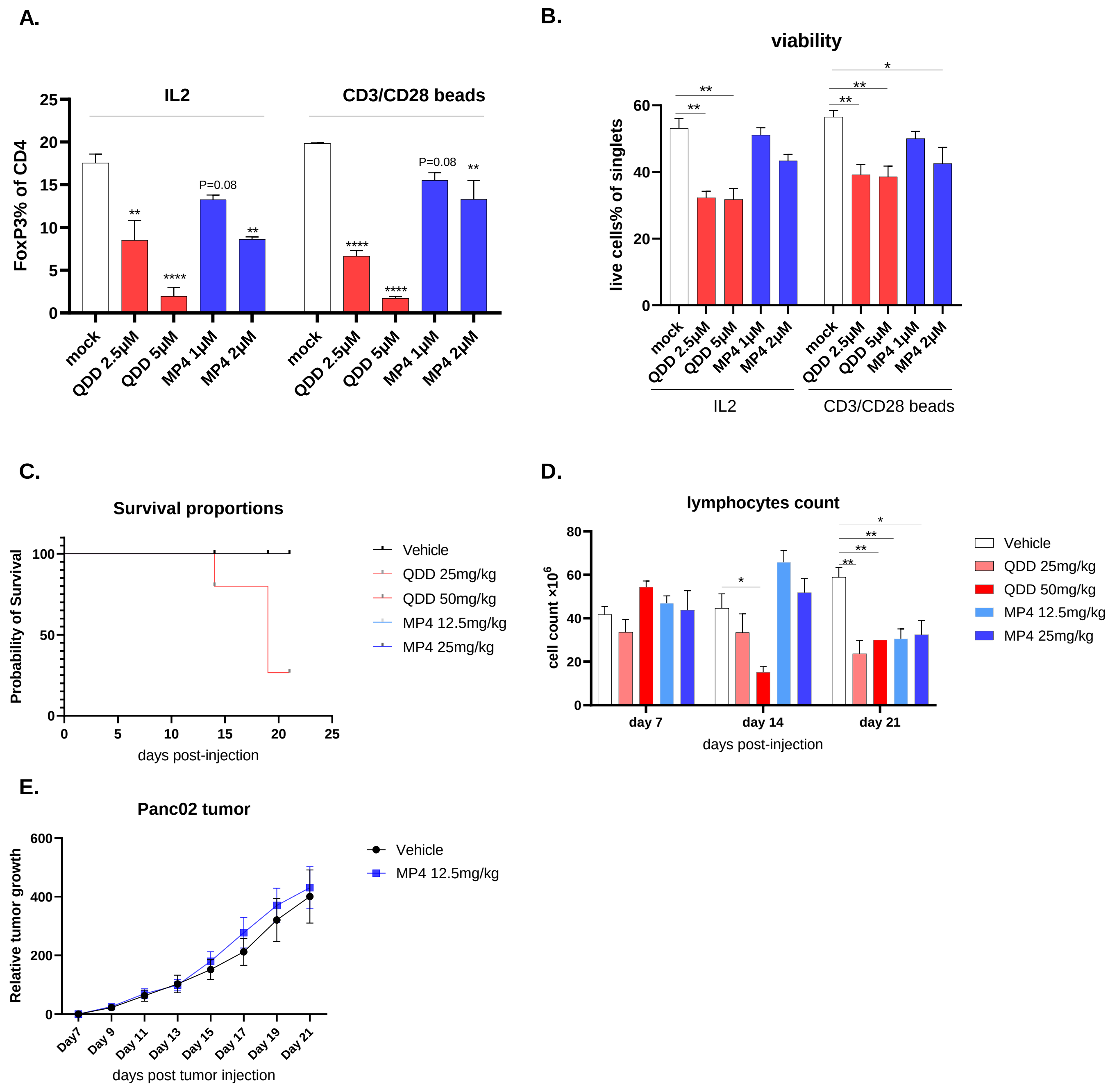

Figure S8. Effects of 9-amino-acridines *in vivo*, related to Figure 6.

(A and B) Splenocytes isolated from WT C57BL/6 mice were treated with QDD or MP4 under IL2 or CD3/CD28 bead stimulation for 48 h. Flow cytometry was then performed to measure FoxP3% in CD4**(A)** and viability**(B)**. **(C and D)** Following the scheme illustrated in Figure 6A, survival proportions of WT C57BL/6 mice treated with indicated compounds for up to 21 days were calculated **(C)**, and total splenocytes numbers at specified time points were counted **(D)**. n=5 mice per group. 2-way ANOVA was used to determine p values. *p<0.05, ** p<0.01. **(E)** Nude mice bearing Panc02 tumors were treated with vehicle, or MP4 (12.5mg/kg) from day seven after tumor cell injection, following the treatment scheme in Figure 6D. Tumor growth curve represents relative tumor size of each time point normalized to that of day seven (n=7 mice per group). Graphs show Mean ±SEM (2-way ANOVA).

Table S1. List of compounds ordered from MolPort, related to Table 1 and Figure 1.

Compound	MolPort ID
MP2	MolPort-004-946-840
MP3	MolPort-005-826-737
MP4	MolPort-000-831-594
MP5	MolPort-001-738-989
MP7	MolPort-006-129-370
MP8	MolPort-006-668-498
MP9	MolPort-001-739-190
MP10	MolPort-005-981-218
MP11	MolPort-003-824-030
MP12	MolPort-039-008-956
MP13	MolPort-000-219-541
MP14	MolPort-002-319-850
MP15	MolPort-002-507-948
MP16	MolPort-001-834-195
MP17	MolPort-000-697-556
MP18	MolPort-001-738-997
MP19	MolPort-001-739-034
MP20	MolPort-001-739-048
MP21	MolPort-001-739-063
MP22	MolPort-001-786-592
MP23	MolPort-002-573-334
MP24	MolPort-023-276-536
MP25	MolPort-008-322-210

Table S2. List of qPCR primers, related to STAR Methods.

	**Forward** (5'-3')	**Reverse** (5'-3')
**FoxP3**	GAACGCCATCCGCCACAACCTGA	CCCTGCCCCCACCACCTCTGC
**FoxP1**	GATTTGCTGTCAGCCATGAA	GGTCACGTCTTACCCCTGAA
**FoxO1**	TTATGACCGAACAGGATGATCTTG	TGTTGGTGATGAGAGAAGGTTGAG
**RPS9**	CGAAGGGTCTCCGCGGGGTCACAT	CGAAGGGTCTCCGCGGGGTCACAT
**CD25**	GAGACTTCCTGCCTCGTCACAA	GAGACTTCCTGCCTCGTCACAA
**CTLA4**	TGCAGCAGTTAGTTCGGGGTTGTT	CTGGCTCTGTTGGGGGCATTTTC
**STAT3**	CTTTGAGACCGAGGTGTATCACC	GGTCAGCATGTTGTACCACAGG
